# Supplementary material for: Schizophrenia-Related Microdeletion Impairs Emotional Memory through MicroRNA-Dependent Disruption of Thalamic Inputs to the Amygdala
Source: Cell Rep. 2017 May 23;19(8):1532–44. doi: 10.1016/j.celrep.2017.05.002 (PMC5457478; doi:10.1016/j.celrep.2017.05.002)
Supplement: Document S1. Supplemental Experimental Procedures, Figures S1–S8, and Table S1 [file mmc1.pdf]

Cell Reports, Volume *19*

Supplemental Information

Schizophrenia-Related Microdeletion Impairs Emotional Memory through MicroRNA-Dependent Disruption of Thalamic Inputs to the Amygdala

Tae-Yeon Eom, Ildar T. Bayazitov, Kara Anderson, Jing Yu, and Stanislav S. Zakharenko

SUPPLEMENTAL EXPERIMENTAL PROCEDURES

Rotarod Test

Motor coordination and balance were assessed using a rotarod (ROTOR-ROD™ System; San Diego Instruments). Mice were placed on a rotating rod (diameter 3.18 cm) in lanes that were 11.4-cm wide to maintain the animal in the same direction while the bar was rotating. The bar was 46 cm from the floor of the apparatus, and the bar's speed of rotation was gradually and linearly increased from 0 to 40 rpm over the 5-min trial. Mice were tested by using five trials per day for 2 consecutive days. Both the latency (s) and the distance (cm) at which the mice were able to maintain their balance on the bar were recorded automatically using the beam break technology.

Hot-plate Test

Nociception was tested using a hot-plate set (Columbus Instruments). Mice were placed on a 55°C hot plate, and the latency period to the first lift of the hind-limb response, including paw shake or licking, was recorded.

In Vivo Viral Injections

For *Drd2* overexpression or knockdown, previously described lentiviruses and AAVs (Chun et al., 2014) were used. Briefly, *AAV-CamKIIα-Drd2-EGFP* and *AAV-CamKIIα-EGFP* were used for the *Drd2* overexpression experiments, and lentiviral vectors *LV-U6-Drd2-EGFP* and *LV-U6-EGFP* were used for the *Drd2*-knockdown experiments. For DREADDs experiments, commercially available *AAV-hSyn-DIO-hM4Di-mCherry* (*hM4Di*) (UNC Vector Core; serotype 5; 6.7 × 10^{12} infection-forming units/mL) and *CAV2-Cre* (Institute of Molecular Genetics of Montpellier, France, and a gift from Dr. Darvas; 6 × 10^{12} particles/mL) were used. For in vivo viral injections, young adult mice were anesthetized with isoflurane in pure oxygen (2%–3% for induction and 1.0%–1.5% for maintenance). Mouse heads were then fixed in a stereotaxic device. Viruses were injected with a metal cannula (33 gauge; Plastics One). Briefly, an incision was made in the scalp, and a small hole was drilled for craniotomy. The following stereotaxic coordinates were used for in vivo injection: MGm (anterior-posterior, –3.16 mm; lateral ±2.0 mm, ventral, –3.0 mm) and LA (anterior-posterior, –1.4 mm; lateral, ±3.4 mm, ventral, –5.0 mm). After injections, incisions were sutured

and mice were allowed to recover before being returned to their holding cages. Experiments were performed approximately 3 weeks after AAV and CAV2 injections and 7 to 10 days after lentiviral injections.

Drug Administration

CNO (Enzo Life Sciences) was dissolved at 0.1 mg/mL in dH_2O and administered via an intraperitoneal injection (1.0 mg/kg) 0.5 h before the start of each behavioral test. Tamoxifen (100 μL, 40 mg/mL; Sigma) was injected intraperitoneally over 2 consecutive days.

Histologic Analysis

At the end of experiments, mice were deeply anesthetized and intracardially perfused with 4% paraformaldehyde in 0.1 mol/L phosphate buffer (pH 7.4), and brains were fixed overnight. Each brain was sliced (50 μm) coronally with a vibratome (Leica) through the rostrocaudal MGm/posterior intralaminar nucleus and the LA and stored in PBS. Virus injection needle tracks were identified with GFP, tdTomato, or mCherry expression, and DAPI (Invitrogen) or bright-field imaging was used as counterstain. Data from mice lacking histologic evidence were excluded from the analysis.

Western Blotting

Mouse brain tissues were lysed in ice-cold RIPA buffer [50 mM Tris-HCl (pH 7.4), 1% NP-40, 0.25% sodium deoxycholate, 150 mM NaCl, and 1 mM EDTA] that included protease and phosphatase inhibitor cocktail tablets (Roche). A total of 25 μg protein was loaded per lane. Sodium dodecyl sulfate/polyacrylamide gel electrophoresis, protein transfer to polyvinylidene difluoride membranes, and Western blotting were performed using standard techniques. The following primary antibodies were used: rabbit anti-DRD2 (Abcam, ab85367, 1:500), anti-DGCR8 (Proteintech, 10996-1-AP, 1:500), and mouse anti–β-actin (Sigma-Aldrich, A5316, 1:10,000). The following secondary antibodies were used: anti-rabbit (LI-COR Biosciences, 926-68021; 1:15,000) and anti-mouse (LI-COR Biosciences, 926-32212, 1:15,000) antibodies conjugated to IR dye 680 or 800, respectively. Blots were imaged and quantified using the Odyssey CLx infrared imaging system (LI-COR Biosciences).

Table S1. Comparison of behavioral and electrophysiological phenotypes in 22q11DS mouse mutants and following disruption of synaptic transmission at thalamoamygdala projections. Related to Figures 1-4, 7.

**Genotype or treatment**[a]	**Fear conditioning (%)**	**Active avoidance**[b] **(%)**	**Basal synaptic transmission**[b] **(%)**	**Paired-pulse depression**[b] **(%)**
Df(16)1/+ mice	–29.4 ± 8.8 *	–26.0 ± 5.8 *	–38.6 ± 7.1 *	–29.4 ± 4.8 *
cDgcr8$^{+/–}$ mice	–23.6 ± 7.9 *	6.3 ± 9.5	–23.5 ± 6.0 *	–10.4 ± 2.7 *
cDgcr8$^{–/–}$ mice	–40.4 ± 7.0 *	–42.3 ± 7.8 *	–65.8 ± 5.2 *	–39.2 ± 2.3 *
Drd2 OE in WT mice	–45.1 ± 7.4 *	–45.7 ± 9.1 *	–77.3 ± 12.8 *	–59.2 ± 5.5 *
Retro-DREADD in WT mice	–26.2 ± 7.0 *	–22.4 ± 8.6 *	–51.9 ± 8.2 *	–33.5 ± 4.5 *

[a] Relative changes for each genotype or treatment group were compared to their respective controls. The *cDgcr8*$^{+/–}$ and *cDgcr8*$^{–/–}$ mice; *Gbx2*^{CreER};*Dgcr8*$^{fl/+}$ and *Gbx2*^{CreER};*Dgcr8*$^{fl/fl}$ mice, respectively. *Drd2* OE: mice with overexpression of *Drd2* in the auditory thalamus. Retro-DREADD: mice injected with *CAV2-Cre* into the lateral amygdala and *hM4Di* into the auditory thalamus and treated with CNO. *$p < 0.05$.

[b]For active avoidance and electrophysiological experiments, values were calculated as normalized to respective WT averages among sessions, stimulating intensities, and interpulse intervals. Data are represented as mean ± SEM.

SUPPLEMENTARY FIGURES

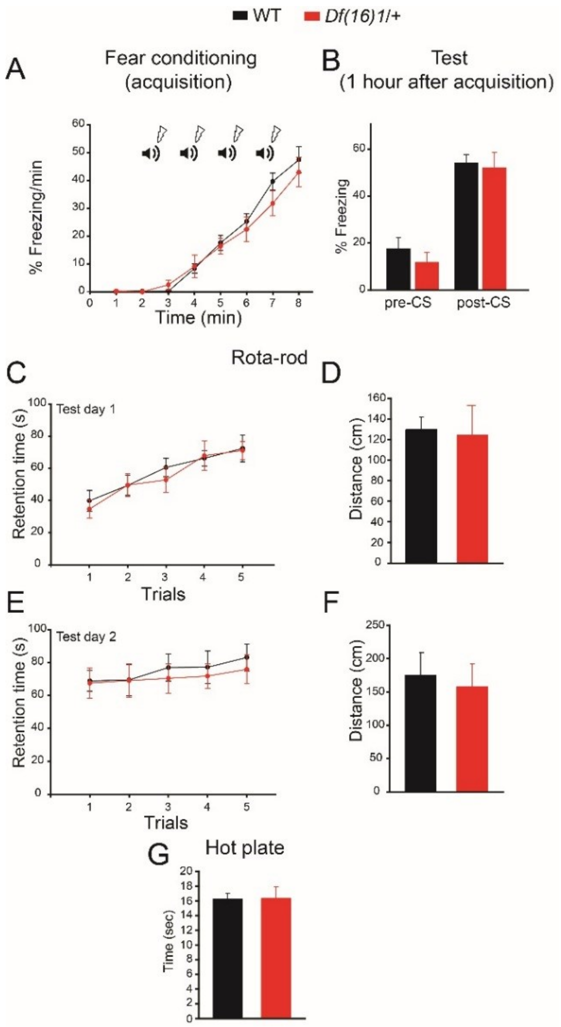

Figure S1. Normal Learning of the Fear Conditioning Task, Locomotion, and Pain Threshold in 22q11DS Mice. Related to Figure 1.

(A) Freezing behavior on training day 1 in WT (n=14) and *Df(16)1*/+ (n=14) animals. Two-way repeated-measures ANOVA; $F_{1,7}$=0.47, p=0.499. Symbols for the conditioned stimuli (CS; tones) and unconditioned stimuli (US; foot shocks) are shown.

(B) Freezing behavior measured in the distinct context 1 hr after fear conditioning in WT and *Df(16)1/*+ mice before tone presentation (pre–CS). WT: 14 mice, *Df(16)1/*+: 14 mice. Mann-Whitney rank sum test: *U*=72.5, p=0.25. Freezing behavior during tone presentation (post-CS): two-tailed Student's *t*-test: t_{26}=0.275, p=0.785.

(C–F) Retention time (C, E) and total distance (D, F) in the rotarod test performed on 2 consecutive days in WT (n=12) and *Df(16)1/*+ (n=11) animals. (C, E) Two-way repeated measures ANOVA. C: $F_{1,4}$=0.131, p=0.721; E: $F_{1,4}$=0.177, p=0.678. (D, F) Mann-Whitney rank sum test. D: *U*=50, p=0.34; F: *U*=56, p=0.559.

(G) Response latency (first lift of the hind limb) in the hot plate test in WT (n=12) and *Df(16)1/*+ (n=11) animals. Mann-Whitney rank sum test: *U*=57.5, p=0.619. Data are represented as mean ± SEM.

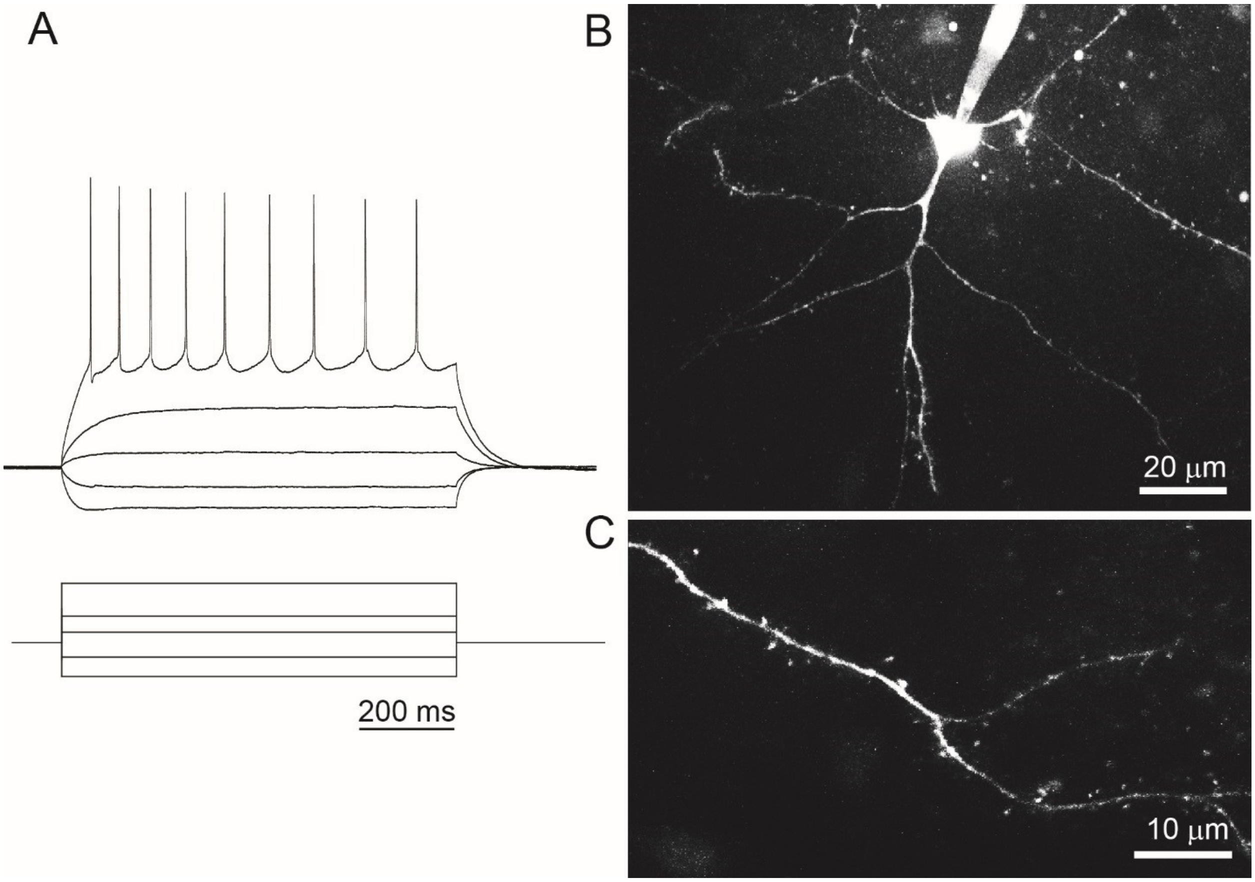

Figure S2. Identification of the Excitatory Neurons in the Lateral Amygdala. Related to Figure 1.
(A) Current-clamp recordings show action potential accommodation in an excitatory LA neuron during the injection of a depolarizing current. (Bottom) A protocol depicting injections of –80 pA, –40 pA, 20 pA, 60 pA, and 140 pA currents into an LA neuron.
(B, C) Fluorescence images of an LA excitatory neuron (B) and its dendrite (C).

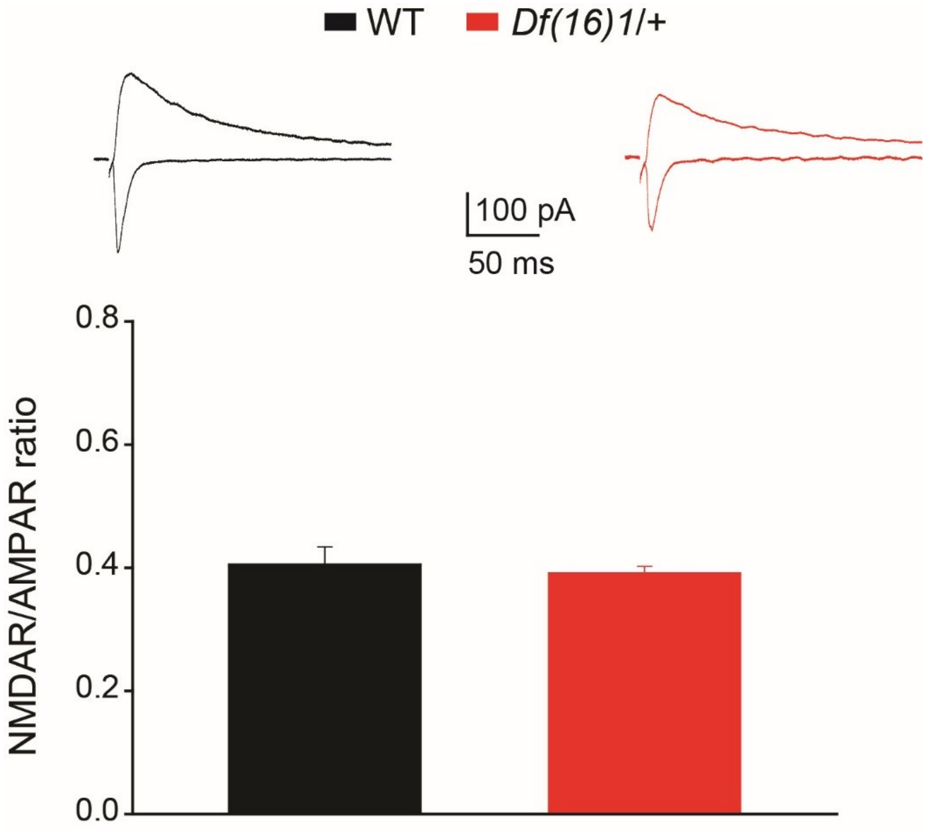

Figure S3. Normal Postsynaptic Function at Thalamo-LA Projections of 22q11DS Mice. Related to Figure 1. Mean NMDAR/AMPAR ratios at thalamo-LA projections of WT (5 neurons, 3 mice) and *Df(16)1/+* (8 neurons, 4 mice) littermates. Two-tailed *t* test: $t_{11}=0.556$, p=0.589. Picrotoxin (100 μM) was present in the ACSF. Insets show representative NMDAR-mediated EPSCs (recorded at +40 mV) and AMPAR-mediated EPSCs (recorded at −70 mV). Data are represented as mean ± SEM.

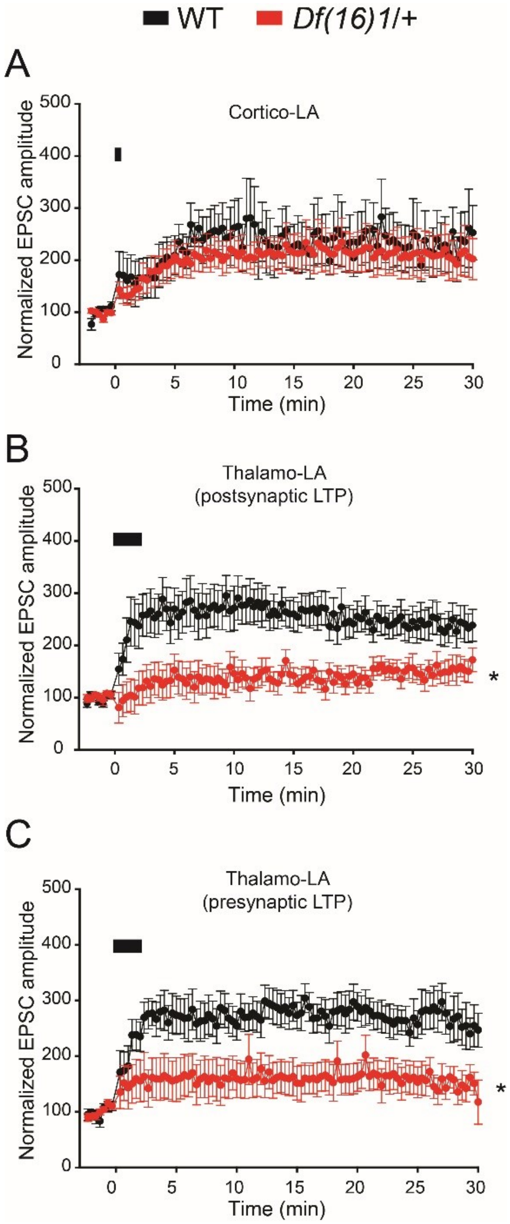

Figure S4. Impaired Thalamo-LA LTP but not Cortico-LA LTP in 22q11DS Mice. Related to Figure 1.

(A-C) Mean excitatory postsynaptic currents (EPSCs) were normalized to initial baseline at cortico-LA (A) and thalamo-LA (B, C) projections, before and after induction of LTP in WT (A: 9 neurons, 3 mice; B: 15 neurons, 8 mice; C: 10 neurons, 5 mice) or *Df(16)1/+* (A: 22 neurons, 8 mice; B: 12 neurons, 5 mice; C: 9 neurons, 5 mice) animals. A: Mann-Whitney rank sum test: U=65, p=0.145, B: two-tailed t test: t_{25}=2.358, *p=0.026, C: two-tailed t test: t_{17}=2.87, *p=0.01. Postsynaptically (B) or presynaptically (C) expressed LTP was induced in thalamo-LA projections by using previously established induction protocols (see Methods) (Shin et al., 2010). Data are represented as mean ± SEM. Solid bars, stimulus-induction protocols.

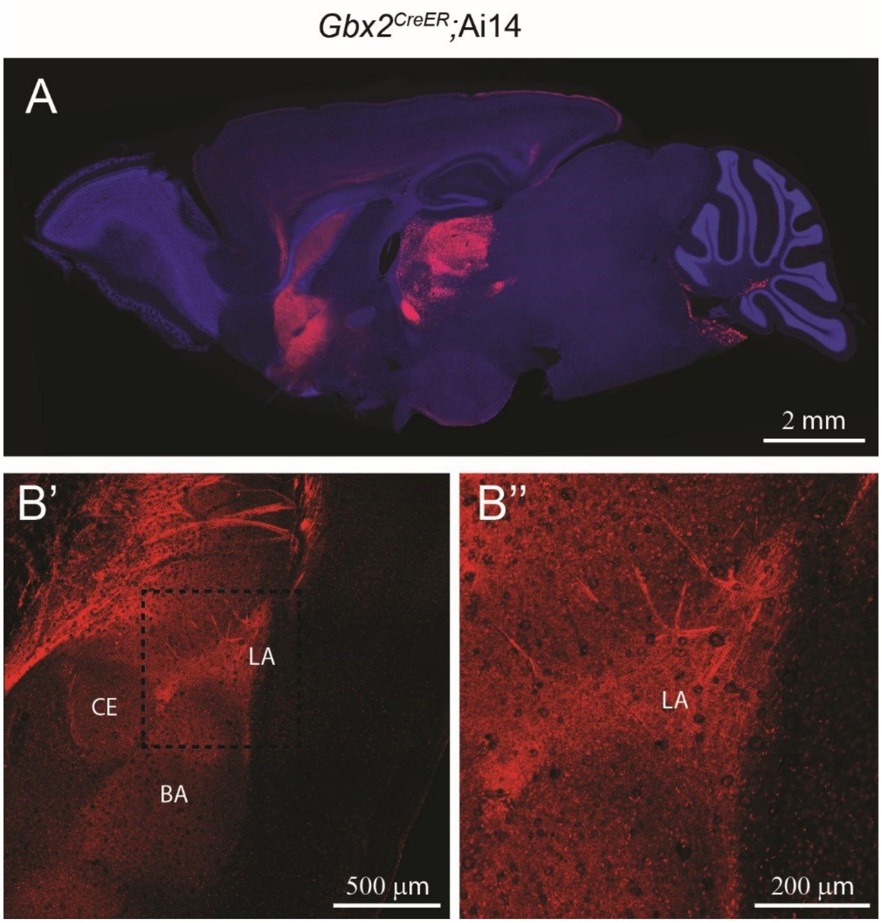

Figure S5. Cre Recombinase Expression in Thalamic Neurons and Their Axonal Projections in *Gbx2^{CreER}*;Ai14 Mice. Related to Figure 2.

(A) Fluorescence image of tdTomato in a sagittal brain section (lateral 0.48 mm) of a mature *Gbx2^{CreER}*;Ai14 mouse 14 days after an intraperitoneal tamoxifen injection. The section is counterstained with DAPI to label nuclei.

(B') The tdTomato-labeled thalamic axonal projections in the LA.

(B'') A higher magnification of the inset in B'. BA, basolateral amygdaloid nucleus; CE, central amygdaloid nucleus; LA, lateral amygdala.

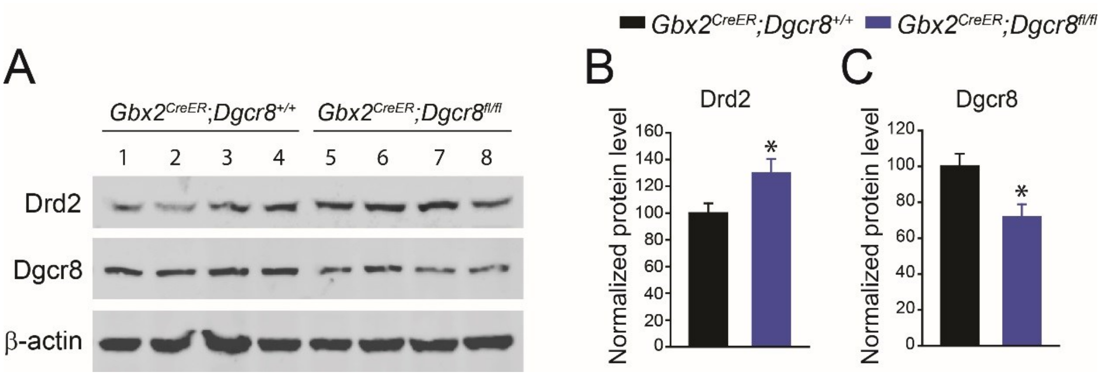

Figure S6. Drd2 and Dgcr8 Protein Levels in the Auditory Thalamus of *cDgcr8* KO Mice. Related to Figure 2.

(A) Representative Western blot images of Drd2 (50 kDa) and Dgcr8 (86 kDa) proteins in the auditory thalamus of WT (*Gbx2*^{CreER};*Dgcr8*$^{+/+}$) and *cDgcr8* KO (*Gbx2*^{CreER};*Dgcr8*$^{fl/fl}$) mice. WT: 4 mice, lanes 1-4; *cDgcr8* KO: 4 mice, lanes 5-8.

(B, C) Relative Drd2 and Dgcr8 protein levels normalized to respective β-actin levels in the auditory thalamus of WT and *cDgcr8* KO animals. Two-tailed *t* test; Drd2 (run in duplicates): t_{14}=2.344, *p=0.034; Dgcr8: t_6=2.805, *p=0.03. Data are represented as mean ± SEM.

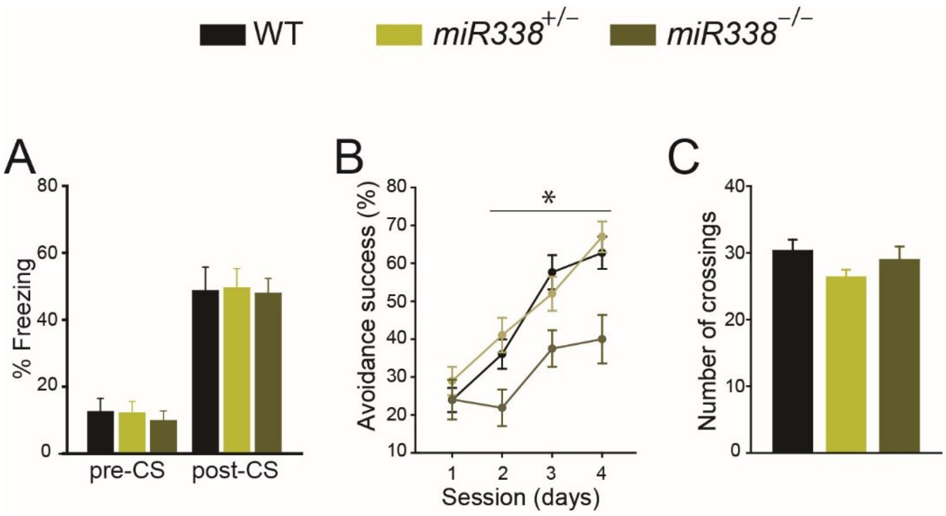

Figure S7. Effect of *miR338* Deletion on Associative Fear Learning. Related to Figure 3.

(A) Freezing behavior in WT (8 mice), *miR338*$^{+/–}$ (13 mice), and *miR-338*$^{–/–}$ (10 mice) littermates during the fear conditioning test. Kruskal-Wallis one-way ANOVA, pre-CS: H_2=0.362, p=0.834; post-CS: F_2=0.003, p=0.978.

(B) Performance of WT (19 mice), *miR338*$^{+/–}$ (20 mice) and *miR-338*$^{–/–}$ (16 mice) littermates in the active avoidance test. Two-way repeated measures ANOVA: $F_{2,3}$=9.940, *p<0.001. Post-hoc: WT vs. *miR338*$^{+/–}$, p=0.593; WT vs. *miR338*$^{–/–}$, *p =0.001.

(C) Total number of crossings between compartments. One-way ANOVA: F_2=1.637, p=0.204. Data are represented as mean ± SEM.

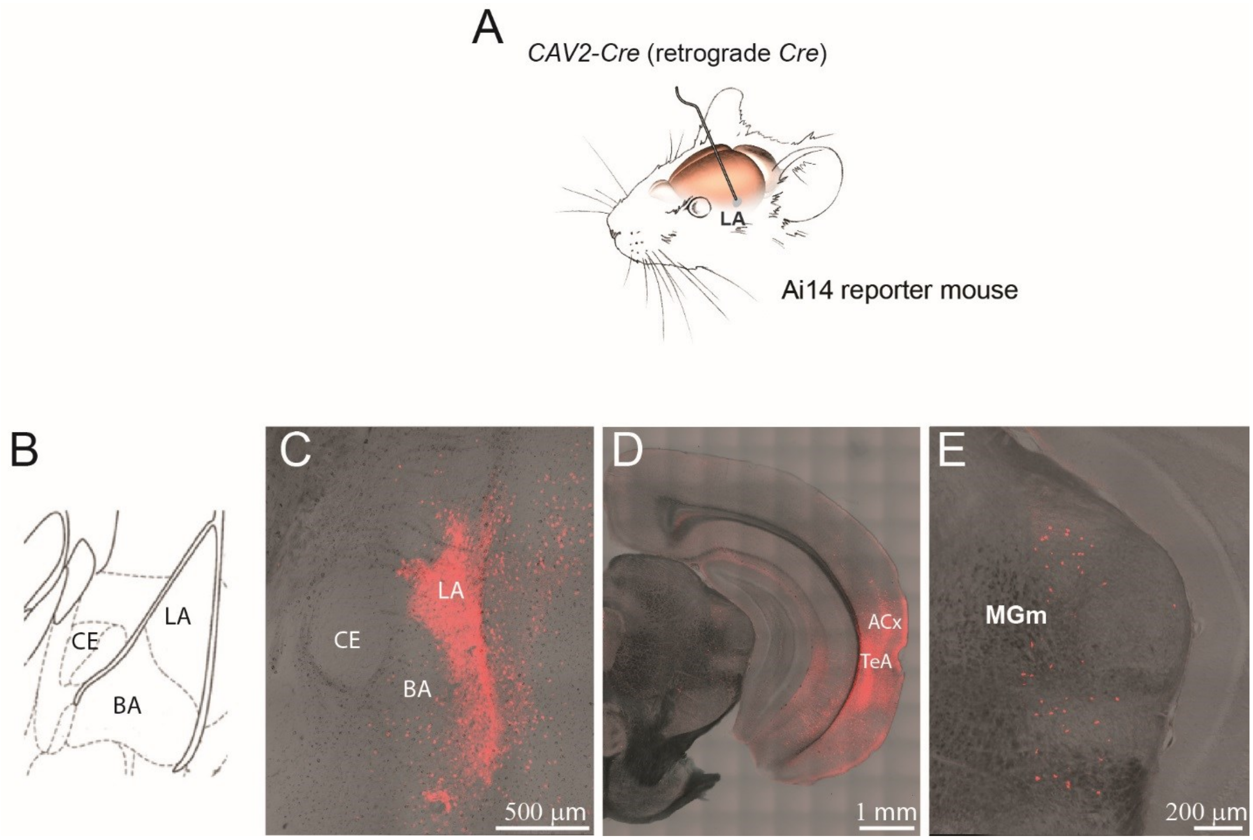

Figure S8. Verification of the Retro-DREADDs Approach in Ai14 Mice. Related to Figure 7.

(A) *CAV2-Cre* viruses were injected into the LA of an Ai14 reporter mouse.

(B) A histologic plate of the amygdaloid nuclei complex in a coronal section at Bregma −1.82 mm, adapted from (Paxinos and Franklin, 2001).

(C–E) Representative images of coronal sections 3 weeks after the *CAV2-Cre* injection into the LA. The expression of tdTomato in the LA depicts the specific targeting of *CAV2–Cre* into the LA (C) and its retrograde infection of neurons in the auditory cortex (ACx), temporal association cortex (TeA) (D), and the auditory thalamus (including the MGm nucleus) (E). BA, basolateral amygdaloid nucleus; CE, central amygdaloid nucleus; LA, lateral amygdala.

Reference List

Chun, S, Westmoreland, JJ, Bayazitov, IT, Eddins, D, Pani, AK, Smeyne, RJ, Yu, J, Blundon, JA, and Zakharenko, SS (2014). Specific disruption of thalamic inputs to the auditory cortex in schizophrenia models. Science. *344*, 1178-1182.

Paxinos,G, and Franklin,KBJ (2001). Mouse Brain in Stereotaxic Coordinates (New York: Academic Press).

Shin, RM, Tully, K, Li, Y, Cho, JH, Higuchi, M, Suhara, T, and Bolshakov, VY (2010). Hierarchical order of coexisting pre- and postsynaptic forms of long-term potentiation at synapses in amygdala. Proc. Natl. Acad. Sci. U. S. A *107*, 19073-19078.
