# Supplementary material for: Schizophrenia-Related Microdeletion Impairs Emotional Memory through MicroRNA-Dependent Disruption of Thalamic Inputs to the Amygdala
Source: Cell Rep. 2017 May 23;19(8):1532–44. doi: 10.1016/j.celrep.2017.05.002 (PMC5457478; doi:10.1016/j.celrep.2017.05.002)
Supplement: Document S2. Article plus Supplemental Information [file mmc2.pdf]

Cell Reports

Article

Schizophrenia-Related Microdeletion Impairs Emotional Memory through MicroRNA-Dependent Disruption of Thalamic Inputs to the Amygdala

Graphical Abstract

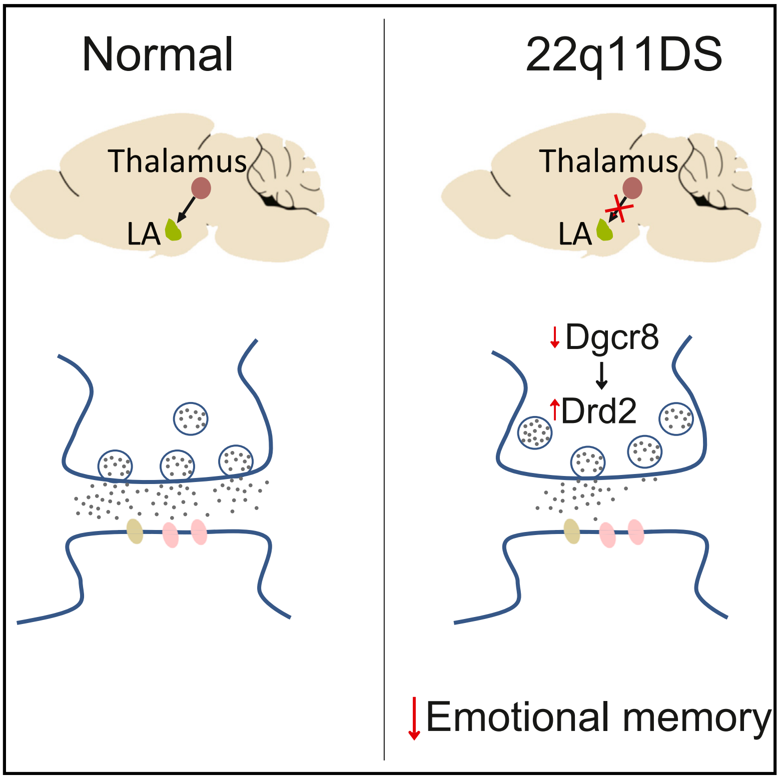

Highlights

- Thalamic inputs to the lateral amygdala (LA) are impaired in 22q11DS mice
- Thalamo-LA disruption is sufficient to cause associative fear memory deficits
- Deficiency in microRNA-processing Dgcr8 causes thalamo-LA and fear memory deficits
- Fear memory deficits in 22q11DS mice are rescued by thalamic Drd2 inhibition

Authors

Tae-Yeon Eom, Ildar T. Bayazitov, Kara Anderson, Jing Yu, Stanislav S. Zakharenko

Correspondence

stanislav.zakharenko@stjude.org

In Brief

Eom et al. show that mouse models of schizophrenia-associated 22q11.2 deletion syndrome are deficient in synaptic transmission at thalamic inputs to the lateral amygdala (LA). Thalamo-LA synaptic deficits impair fear memory and are mediated by *Dgcr8* haploinsufficiency and *Drd2* elevation in the auditory thalamus.

Eom et al., 2017, Cell Reports *19*, 1532–1544
May 23, 2017 © 2017 The Author(s).
http://dx.doi.org/10.1016/j.celrep.2017.05.002

OPEN ACCESS
CellPress

Cell Reports
Article

Schizophrenia-Related Microdeletion Impairs Emotional Memory through MicroRNA-Dependent Disruption of Thalamic Inputs to the Amygdala

Tae-Yeon Eom,[1] Ildar T. Bayazitov,[1] Kara Anderson,[1] Jing Yu,[1] and Stanislav S. Zakharenko[1,2,*]
[1]Department of Developmental Neurobiology, St. Jude Children's Research Hospital, Memphis, TN 38105, USA
[2]Lead Contact
*Correspondence: stanislav.zakharenko@stjude.org
http://dx.doi.org/10.1016/j.celrep.2017.05.002

SUMMARY

Individuals with 22q11.2 deletion syndrome (22q11DS) are at high risk of developing psychiatric diseases such as schizophrenia. Individuals with 22q11DS and schizophrenia are impaired in emotional memory, anticipating, recalling, and assigning a correct context to emotions. The neuronal circuits responsible for these emotional memory deficits are unknown. Here, we show that 22q11DS mouse models have disrupted synaptic transmission at thalamic inputs to the lateral amygdala (thalamo-LA projections). This synaptic deficit is caused by haploinsufficiency of the 22q11DS gene *Dgcr8*, which is involved in microRNA processing, and is mediated by the increased dopamine receptor Drd2 levels in the thalamus and by reduced probability of glutamate release from thalamic inputs. This deficit in thalamo-LA synaptic transmission is sufficient to cause fear memory deficits. Our results suggest that dysregulation of the Dgcr8–Drd2 mechanism at thalamic inputs to the amygdala underlies emotional memory deficits in 22q11DS.

INTRODUCTION

Emotions provide information about the present state of an individual based on previous experience and help guide future courses of action. In healthy individuals, many action-related decisions are thought to be based on anticipation or recall of emotional experiences (Wilson and Gilbert, 2003). Past positive or negative emotional experiences can help decide whether the same action should be repeated in the future. In contrast, patients with schizophrenia (SCZ) are impaired in their ability to anticipate or recall emotions (Engel et al., 2015; Gard et al., 2007; Kring and Elis, 2013). These deficits in emotional memory can be caused by impairments in memory consolidation processes for emotional stimuli and may contribute to the negative symptoms of SCZ, such as anhedonia or amotivation (Herbener et al., 2007). Previous studies have found a relationship between emotional memory deficits and negative symptoms (Hall et al., 2007; Herbener, 2008; Horan et al., 2006). However, the neuronal circuits that underlie such deficits in emotional memory are not yet known.

Insights into identifying the neuronal circuits responsible for deficits in SCZ-related emotional memory can be gained using murine models of 22q11.2 deletion syndrome (22q11DS), which is the most common microdeletion syndrome in humans (Bassett et al., 2011; McDonald-McGinn and Sullivan, 2011). Patients with 22q11DS have a significant predisposition for SCZ (Bassett and Chow, 1999; Gothelf et al., 1999; Green et al., 2009; Murphy et al., 1999; Murphy, 2002; Pulver et al., 1994; Shprintzen et al., 1992). This syndrome is caused by the hemizygous deletion of a 1.5- to 3-Mb region of the q arm of chromosome 22, which results in the deletion of one copy of more than 20 genes (Scambler et al., 1992). The 22q11DS-critical region is largely conserved on mouse chromosome 16, which allows the generation of 22q11DS mouse models (*Df(16)1/+*) carrying a hemizygous deletion of 23 genes in the syntenic region of chromosome 16 (Lindsay et al., 1999). The symptoms of 22q11DS-related SCZ are indistinguishable from those of the idiopathic disease (Chow et al., 2006; Murphy et al., 1999; Pulver et al., 1994). Emotional deficits occur in patients with 22q11DS (Campbell et al., 2006; Leleu et al., 2016; Shprintzen, 2000), such as impairments in facial memory (Lajiness-O'Neill et al., 2005) and difficulty in recognizing facial expressions of emotions, especially anger, disgust, and fear (Campbell et al., 2010; McCabe et al., 2011). These impairments may contribute to emotional memory deficit similar to that seen in patients with SCZ.

Previous studies on 22q11DS mice narrowed down several SCZ-related phenotypes to haploinsufficiency of the microRNA biogenesis gene *Dgcr8* (Chun et al., 2014, 2017; Earls et al., 2012; Stark et al., 2008). DGCR8 binds primary microRNA transcripts and recruits the nuclease DROSHA to cleave transcripts. Further processing yields mature microRNAs that bind to complementary seed sites in the 3′ UTRs of target mRNA transcripts and negatively regulate the stability of the target transcript or protein translation (Bartel, 2009). We previously reported that the deletion of one copy of *Dgcr8* impairs glutamatergic synaptic transmission at thalamic inputs to the auditory cortex by increasing the levels of dopamine receptors D2 (Drd2s) in the thalamus (Chun et al., 2014).

Thalamic neurons send projections to the lateral amygdala (LA), which is part of the basolateral amygdala that is important

 © 2017 The Author(s).
This is an open access article under the CC BY license (http://creativecommons.org/licenses/by/4.0/).

for assigning emotional significance to discrete environmental cues and acquiring and storing emotional memories (LeDoux, 2003; Maren and Quirk, 2004; Rosenkranz and Grace, 2002). In rodents, the acquisition and expression of aversive memories are conventionally studied with the Pavlovian fear (threat) conditioning (LeDoux, 2000) or active avoidance training paradigms (Cain and LeDoux, 2008), though these methods are different from those used to probe emotional deficits in individuals with SCZ (Engel et al., 2015; Gard et al., 2007; Kring and Elis, 2013). Rodents are trained to associate an environmental cue (a conditioned stimulus [CS]), such as a sound, with an aversive cue (an unconditioned stimulus [US]), such as an electrical footshock. LA neurons are the first site of convergence of sensory inputs carrying CS and US information to the amygdala (Azuma et al., 1984; LeDoux, 2000; Nakashima et al., 2000). Thalamo-amygdala (thalamo-LA) projections and cortico-amygdala (cortico-LA) projections convey CS information to the LA, and synaptic plasticity at thalamo-LA projections is involved in emotional memory (Cho et al., 2011; McKernan and Shinnick-Gallagher, 1997; Rogan et al., 1997; Rumpel et al., 2005; Tye et al., 2008). In the auditory fear conditioning task, CS inputs are carried by thalamo-LA and cortico-LA projections arising from the auditory thalamus and auditory cortex, respectively (Quirk et al., 1995, 1997; Romanski and LeDoux, 1992). Pavlovian fear conditioning results in animal freezing upon delivering the CS. Active avoidance behavior depends on Pavlovian information but also requires instrumental learning to suppress freezing and keep it attenuated when the CS is presented to enable animals to perform the avoidance response (Cain and LeDoux, 2008). Although the behavioral outputs of these two tests are different, both depend on the delivery of the CS to the LA.

Because we have previously shown that microdeletion of 22q11DS genes increases Drd2 levels in the auditory thalamus (Chun et al., 2014) and that the auditory thalamus is important for the delivery of the CS to the amygdala—and thus for emotional memory—we hypothesized that fear memory is impaired in 22q11DS mice and that this deficit could be due to Dgcr8–Drd2-dependent impairment of synaptic transmission at thalamo-LA projections. We also hypothesized that inhibition or reduction of Drd2s in the auditory thalamus rescues deficits in fear memory in 22q11DS mice.

RESULTS

Fear Memory and Thalamo-LA Synaptic Transmission and Plasticity Are Impaired in 22q11DS Mice

To test our hypothesis, we used mature (4- to 5-month-old) *Df(16)1*/+ mouse models of 22q11DS (*Df(16)1*/+ mice) (Figure 1A). We tested associative fear memory by the fear conditioning and active avoidance tasks. Both tests indicated that fear memory is impaired in *Df(16)1*/+ mice (Figures 1B–1D; Table S1). In the fear conditioning task, CS-induced freezing was recorded 1 or 24 hr after training in a distinct context. During the training session, *Df(16)1*/+ mice and wild-type (WT) mice did not differ in their freezing responses to the CS–US pairings (Figure S1A) or during the CS presentation 1 hr after training (Figure S1B). However, 24 hr after training, the CS presentation caused significantly less freezing in *Df(16)1*/+ mice, compared to WT littermates, but no significant differences were seen in pre-CS freezing between the genotypes (Figure 1C). These results suggested that *Df(16)1*/+ mice have a deficit in the retrieval of auditory cued fear memory but not in the acquisition. In the active avoidance task, *Df(16)1*/+ mice exhibited a significantly lower percentage of escape success than did WT littermates (Figure 1D). However, the total number of spontaneous crossings between compartments was not different between the genotypes, suggesting that locomotor activity, motor coordination, and balance were not affected in *Df(16)1*/+ mice (Figure 1E). This finding was confirmed by the rotarod test (Figures S1C–S1F). Sensitivity to pain measured in the hot-plate test was also comparable between the genotypes (Figure S1G).

To explore whether the mechanisms of synaptic transmission and plasticity at thalamo-LA and cortico-LA projections are affected in mutant mice, we performed whole-cell voltage-clamp recordings from excitatory neurons in the LA in acute brain slices from both groups of animals (Figure 1F). Recorded neurons exhibited strong action potential accommodation and possessed dendritic spines (Figure S2), suggesting that they are principal neurons. Thalamo-LA and cortico-LA projections were activated independently by placing stimulation electrodes onto the internal capsule or the external capsule, respectively (Cho et al., 2011; Mahanty and Sah, 1998; Tsvetkov et al., 2002).

To assay the effects of the 22q11.2 deletion on synaptic transmission, we compared input–output relations at cortico-LA and thalamo-LA projections between mutant and WT mice. Excitatory postsynaptic currents (EPSCs) at cortical inputs were not different between the genotypes (Figure 1G), but the synaptic strength at thalamic inputs to the LA was substantially reduced in *Df(16)1*/+ mice, compared to WT littermates (Figure 1H; Table S1). The paired-pulse ratio (PPR; an index of presynaptic function) (Zucker and Regehr, 2002) in cortical input to the LA was identical between the genotypes (Figure 1I). However, paired-pulse depression (PPD; a feature of thalamic inputs that is measured by PPR) (Bayazitov et al., 2013; Blundon et al., 2011; Chun et al., 2013; Gil et al., 1999; Viaene et al., 2011) was lower in *Df(16)1*/+ mice, compared to WT littermates (Figure 1J; Table S1), indicating that synaptic deficits in thalamo-LA projections in *Df(16)1*/+ mice might be presynaptic in origin. The thalamo-LA NMDAR/AMPAR (NMDA receptor/AMPA receptor) ratio, a measure of the postsynaptic function in thalamic inputs to LA, was normal in *Df(16)1*/+ mice (Figure S3). However, long-term potentiation (LTP) was impaired in thalamo-LA projections of *Df(16)1*/+ mice, whereas there was no difference in the magnitude of LTP at cortico-LA synapses between the genotypes (Figure S4).

Conditional Ablation of *Dgcr8* in Thalamic Neurons Mimics Thalamo-LA Synaptic and Behavioral Deficits of 22q11DS Mice

To test the role of *Dgcr8* in these phenotypes, we deleted it in thalamic neurons by crossing mice with the floxed *Dgcr8* allele (Wang et al., 2007) with *Gbx2*^{CreER} mice (Chen et al., 2009) that express Cre recombinase in thalamic neurons (Chatterjee

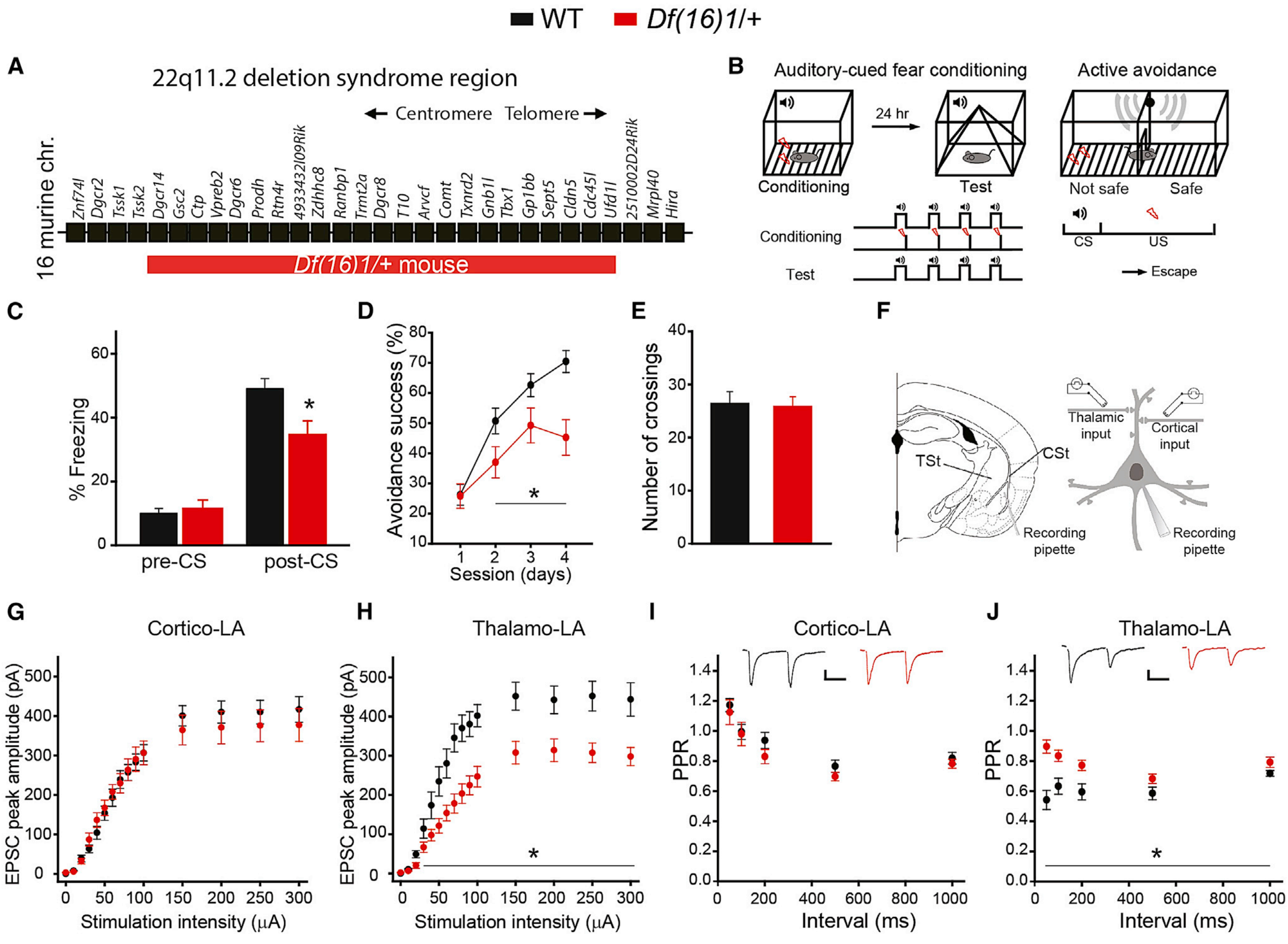

Figure 1. Associative Fear Memory and Thalamo-LA Synaptic Transmission Are Impaired in 22q11DS Mice

(A) Map of 22q11DS orthologs deleted in *Df(16)1*/+ mice. chr, chromosome.

(B) Schematics for the auditory cued fear conditioning and active avoidance tasks.

(C) Freezing behavior in the distinct context 24 hr after fear conditioning in WT and *Df(16)1*/+ mice before tone presentation (pre-conditioned stimulus [CS]). WT: 16 mice, *Df(16)1*/+: 13 mice. Mann-Whitney rank-sum test; U = 193, p = 0.948. Freezing behavior during tone presentation (post-CS): two-tailed Student's t test; t(27) = 2.76; *p = 0.01.

(D) Active avoidance success rates as a function of the number of training days in WT and *Df(16)1*/+ mice. WT, 21 mice; *Df(16)1*/+, 20 mice. Two-way repeated-measures ANOVA, F(1, 3) = 11.292; *p = 0.002.

(E) Total number of crossings between compartments in the active avoidance task (WT, 21 mice; *Df(16)1*/+, 20 mice; two-tailed Student's t test; t_{39} = 0.199, p = 0.843).

(F) Positions of whole-cell voltage-clamp recordings (recording pipette) from LA neurons and placements of stimulation electrodes for activating thalamic (TSt) and cortical (CSt) inputs.

(G and H) Excitatory postsynaptic current (EPSC) peak amplitude as a function of stimulation intensity at cortico-LA (G) and thalamo-LA (H) projections in WT mice (19 neurons, four mice) and *Df(16)1*/+ mice (22 neurons, five mice). Two-way repeated-measures ANOVA: in (G), $F_{1,14}$ = 0.18, p = 0.673; in (H), $F_{1,14}$ = 13.85, *p < 0.001.

(I and J) Paired-pulse ratio (PPR) at cortico-LA (I) and thalamo-LA (J) projections in WT mice (I: 15 neurons, three mice; J: 14 neurons, three mice) and *Df(16)1*/+ mice (I: 15 neurons, three mice; J: 14 neurons, five mice). Two-way repeated-measures ANOVA: in (I: $F_{1,4}$ = 1.06, p = 0.311; in (J), F(1, 4) = 15.58, *p < 0.001. Insets, representative pairs of cortico-LA and thalamo-LA EPSPs, respectively. Scale bars, 50 pA, 50 ms. Data are represented as mean ± SEM.

See also Figures S1–S4.

et al., 2012) after tamoxifen induction. Mice with the conditional deletion of *Dgcr8* (*cDgcr8* KO [knockout]) (*Gbx2*^{CreER};*Dgcr8*$^{fl/+}$ and *Gbx2*^{CreER};*Dgcr8*$^{fl/fl}$) developed normally and had no gross morphologic abnormalities (data not shown). To verify Cre expression, we crossed *Gbx2*^{CreER} mice with Ai14 reporter mice (*ROSA26-CAG-Stop*$^{fl/fl}$*-tdTomato*) (Madisen et al., 2010). Fourteen days after the tamoxifen injection, there was strong tdTomato expression in the thalamus of *Gbx2*^{CreER};Ai14 mice (Figures 2A and S5). A qRT-PCR analysis confirmed lower levels of the *Dgcr8* transcript in the auditory thalamus of *cDgcr8* KO mice than in that of the WT littermates. This reduction was dose dependent; that is, heterozygous and homozygous *Dgcr8*

OPEN ACCESS CellPress

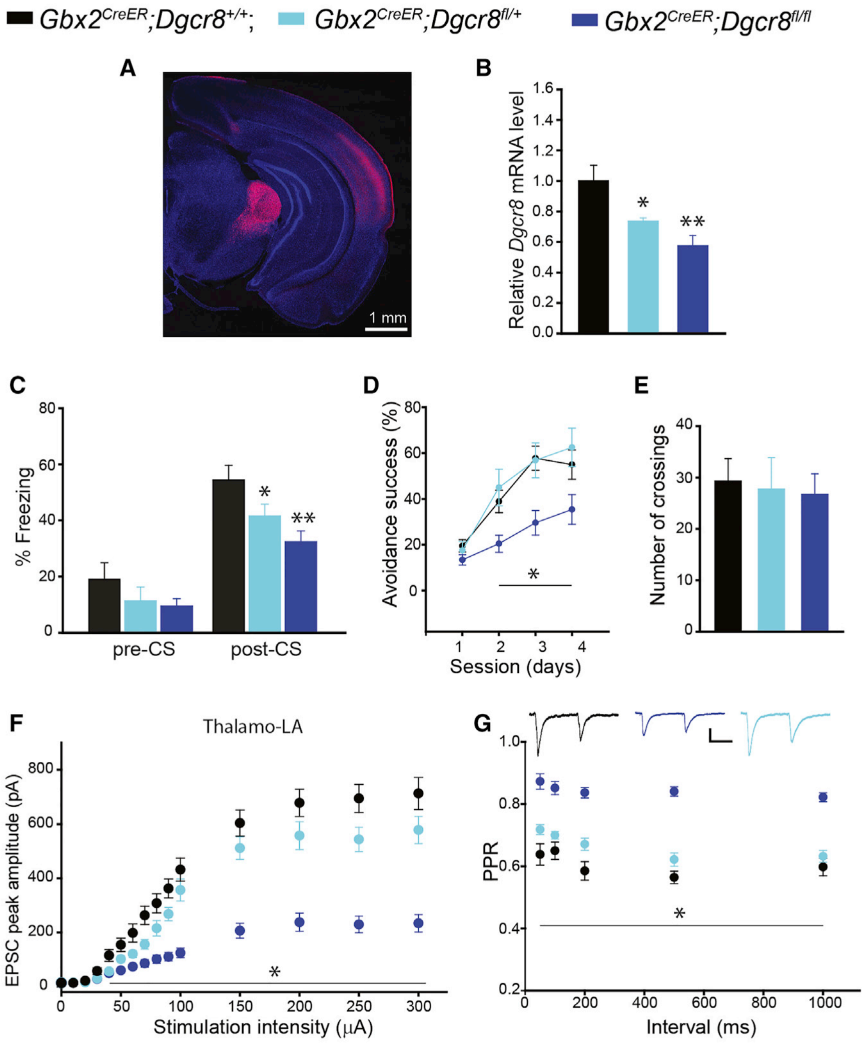

Figure 2. Thalamus-Specific *Dgcr8* Deletion Impairs Associative Fear Memory and Synaptic Transmission at Thalamo-LA Projections

(A) Expression of tdTomato in a coronal brain section of $Gbx2^{CreER}$;*Ai14* mouse.

(B) *Dgcr8* transcript levels in the auditory thalamus of WT ($Gbx2^{CreER}$;$Dgcr8^{+/+}$) and *cDgcr8* KO ($Gbx2^{CreER}$;$Dgcr8^{fl/+}$ and $Gbx2^{CreER}$;$Dgcr8^{fl/fl}$) mice injected with tamoxifen (WT, four mice; $Gbx2^{CreER}$;$Dgcr8^{fl/+}$, three mice; $Gbx2^{CreER}$;$Dgcr8^{fl/fl}$, three mice). Mann-Whitney rank-sum test: WT versus $Gbx2^{CreER}$;$Dgcr8^{fl/+}$, U = 73.5, *p = 0.042; WT versus $Gbx2^{CreER}$;$Dgcr8^{fl/fl}$, U = 37, **p = 0.007. Experiments were performed in triplicate.

(C) Freezing behavior before (pre-CS) and during (post-CS) presentation of the CS in WT and *cDgcr8* KO mice. Pre-CS (WT, 11 mice; $Gbx2^{CreER}$;$Dgcr8^{fl/+}$, 8 mice; $Gbx2^{CreER}$;$Dgcr8^{fl/fl}$, 11 mice). Kruskal-Wallis one-way ANOVA, H_2 = 1.724; p = 0.422. Post-CS: one-way ANOVA, F_2 = 6.348, *p = 0.006. WT versus $Gbx2^{CreER}$;$Dgcr8^{fl/+}$: t_{17} = 1.779, *p = 0.05; WT versus $Gbx2^{CreER}$;$Dgcr8^{fl/fl}$: t_{20} = 3.372, **p = 0.003.

(D) Performance of WT and *cDgcr8* KO mice in the active avoidance (WT, 9 mice; $Gbx2^{CreER}$;$Dgcr8^{fl/+}$, 8 mice; $Gbx2^{CreER}$;$Dgcr8^{fl/fl}$, 12 mice). Two-way repeated-measures ANOVA: F(2, 3) = 10.113, p < 0.001. For WT versus $Gbx2^{CreER}$;$Dgcr8^{fl/+}$, p = 0.634; for WT versus $Gbx2^{CreER}$;$Dgcr8^{fl/fl}$, *p = 0.003.

(E) Total number of crossings between compartments. The same number of mice was used as in (D). One-way ANOVA: F_2 = 0.08, p = 0.923.

(F) EPSC peak amplitude as a function of stimulation intensity at thalamo-LA projections in WT and *cDgcr8* KO mice (WT: 16 neurons, four mice; $Gbx2^{CreER}$;$Dgcr8^{fl/+}$: 15 neurons, four mice; $Gbx2^{CreER}$;$Dgcr8^{fl/fl}$: 20 neurons, six mice). Two-way repeated-measures ANOVA: F(2, 14) = 37.4, *p < 0.001. Post hoc: WT versus $Gbx2^{CreER}$;$Dgcr8^{fl/+}$, p = 0.007; WT versus $Gbx2^{CreER}$;$Dgcr8^{fl/fl}$, p < 0.001.

(G) PPR at thalamo-LA projections in WT and *cDgcr8* KO mice. (WT: 18 neurons, four mice; $Gbx2^{CreER}$;$Dgcr8^{fl/+}$: 18 neurons, four mice; $Gbx2^{CreER}$;$Dgcr8^{fl/fl}$: 22 neurons, six mice). Two-way repeated-measures ANOVA: F(2, 4) = 69.9, *p < 0.001. Post hoc: WT versus $Gbx2^{CreER}$;$Dgcr8^{fl/+}$, p = 0.008; WT versus $Gbx2^{CreER}$;$Dgcr8^{fl/fl}$, p < 0.001. Insets show representative pairs of thalamo-LA EPSCs in WT and *cDgcr8* KO mice. Scale bars, 100 pA, 50 ms. Data are represented as mean ± SEM.

See also Figures S5 and S6.

deletions reduced *Dgcr8* mRNA levels by 26.3% ± 2.1% and 42.4% ± 6.6%, respectively (Figure 2B).

Similar to 22q11DS mice, *cDgcr8* KO mice had impaired fear conditioning (Table S1). In mutant animals, the deficiency in auditory cued fear conditioning occurred in a dose-dependent manner, whereas there was no effect on the pre-CS freezing level (Figure 2C). The *cDgcr8* KO mice also had impaired active avoidance (Figure 2D; Table S1). This deficit occurred in homozygous, but not heterozygous, *cDgcr8* KO mice. Conditional deletion of *Dgcr8* did not affect locomotor activity (Figure 2E).

Thalamo-LA EPSCs were smaller in *cDgcr8* KO mice than in WT littermates (Figure 2F). Notably, homozygous deletion of *Dgcr8* in the thalamus reduced thalamo-LA EPSCs by approximately 65% (Table S1). As seen in *Df(16)1/+* mice, PPD was also reduced in *cDgcr8* KO mice (Figure 2G; Table S1).

Knockdown of Drd2 in the Thalamus Rescues Associative Fear Memory and Thalamo-LA Synaptic Transmission Deficits in 22q11DS Mice

Because deletion of *Dgcr8* in the thalamus resembles the thalamo-LA synaptic and fear memory deficits of *Df(16)1/+* mice, we next asked whether these deficits are mediated by elevated levels of Drd2 in the thalamus. We observed a decrease in Dgcr8 protein level, accompanied by an increase in Drd2 in the auditory thalamus of *cDgcr8* KO mice (Figure S6). To further examine the effect of Drd2, we knocked down Drd2 in the auditory thalamus through in vivo injections of lentiviruses encoding short hairpin RNA (shRNA) against *Drd2* (*Drd2* shRNA) and GFP in the medial division of the medial geniculate nucleus (MGm) (Figure 3A). The specificity of these shRNAs against Drd2 was previously confirmed (Chun et al.,

OPEN ACCESS
CellPress

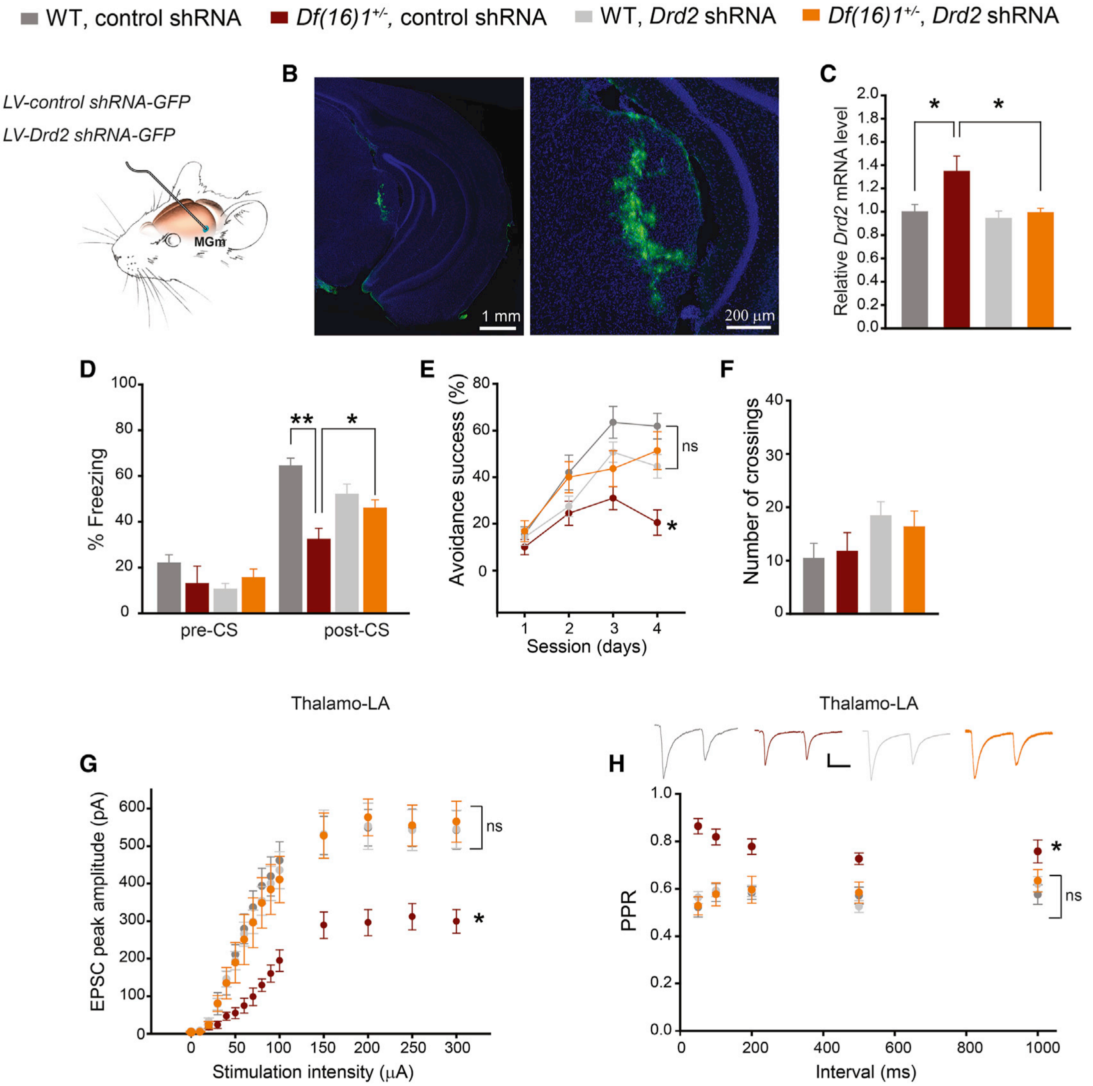

Figure 3. *Drd2* Knockdown in the Auditory Thalamus Rescues Deficits in Associative Fear Memory and Thalamo-LA Synaptic Transmission in 22q11DS Mice

(A) In vivo infection of MGm neurons with lentiviral vector *LV-control shRNA-GFP* (control shRNA) or *LV-Drd2 shRNA-GFP* (*Drd2* shRNA).

(B) Representative images of a coronal brain section from a mouse infected with control shRNA showing GFP$^+$ neurons in the MGm under low (left) and high (right) magnifications. Sections were counterstained with DAPI (nuclei).

(C) Relative *Drd2* mRNA levels in WT and *Df(16)1*/+ mice injected with control or *Drd2* shRNA (WT injected with control shRNA, six mice; *Df(16)1*/+ injected with control shRNA, six mice; WT injected with *Drd2* shRNA, five mice; *Df(16)1*/+ injected with *Drd2* shRNA, five mice). Mann-Whitney rank-sum test: for WT injected with control shRNA versus *Df(16)1*/+ injected with control shRNA, U = 5, *p = 0.041; for *Df(16)1*/+ injected with control shRNA versus *Df(16)1*/+ injected with *Drd2* shRNA, U = 4, *p = 0.026.

(D) Freezing behavior before (pre-CS) and during (post-CS) presentation of the CS in WT and *Df(16)1*/+ mice injected with control or *Drd2* shRNAs (WT injected with control shRNA, 11 mice; *Df(16)1*/+ injected with control shRNA, 10 mice; WT injected with *Drd2* shRNA, 14 mice; *Df(16)1*/+ injected with *Drd2* shRNA, 14 mice). Pre-CS: Kruskal-Wallis one-way ANOVA on ranks, $H_3 = 7.677$, p = 0.53. Post-CS: one-way ANOVA, $F_3 = 8.786$, *p < 0.001. Post hoc: *Df(16)1*/+ injected with control shRNA versus *Df(16)1*/+ injected with *Drd2* shRNA, *p = 0.033; WT injected with control shRNA versus *Df(16)1*/+ injected with control shRNA, **p = 0.000013.

(E) Active avoidance performance in WT and *Df(16)1*/+ mice injected with control or *Drd2* shRNAs (WT injected with control shRNA, 10 mice; *Df(16)1*/+ injected with control shRNA, 10 mice; WT injected with *Drd2* shRNA, 14 mice; *Df(16)1*/+ injected with *Drd2* shRNA, 11 mice). Two-way repeated-measures ANOVA: $F_{3,3} = 7.392$, *p < 0.001. Post hoc at day 4: *Df(16)1*/+ injected with control shRNA versus *Df(16)1*/+ injected with *Drd2* shRNA, *p = 0.006; WT injected with control shRNA versus *Df(16)1*/+ injected with *Drd2* shRNA, p = 0.338.

(F) Total number of crossings between compartments in WT and *Df(16)1*/+ mice injected with control or *Drd2* shRNAs. The same number of mice as used in (E). One-way ANOVA: $F_3 = 1.626$, p = 0.198.

(G) EPSC peak amplitude, as a function of stimulation intensity at thalamo-LA projections of the following groups are shown: WT injected with control shRNA (11 neurons, three mice), *Df(16)1*/+ injected with control shRNA (13 neurons, three mice), WT injected with *Drd2* shRNA (13 neurons, three mice), and *Df(16)1*/+ injected with *Drd2* shRNA (9 neurons, three mice). Two-way repeated-measures ANOVA: $F_{3,14} = 8.673$, p < 0.001. Post hoc: *p < 0.01.

(legend continued on next page)

OPEN ACCESS
CellPress

2014). Neurons in the auditory thalamus were successfully infected by in vivo injections of these viruses (Figure 3B). Note that some neurons outside the auditory thalamus were also infected. In mice injected with control shRNA, *Drd2* transcript levels were higher in *Df(16)1*/+ mice than in WT littermates (Figure 3C). This increase in Drd2 in *Df(16)1*/+ mice was rescued by *Drd2* shRNAs, which decreased the *Drd2* mRNA level in mutant mice to that in WT littermates. In WT littermates, *Drd2* shRNAs did not affect *Drd2* mRNA levels in the auditory thalamus (Figure 3C).

Drd2 shRNA did not affect pre-CS freezing in WT or *Df(16)1*/+ mice. However, *Drd2* shRNA rescued the deficit in 22q11DS mice in the fear conditioning task (Figure 3D). *Df(16)1*/+ mice injected with *Drd2* shRNA performed significantly better than did *Df(16)1*/+ mice injected with control shRNA. Moreover, the performance in fear conditioning was similar in *Df(16)1*/+ and WT mice injected with Drd2 shRNA (Figure 3D). Drd2 shRNA also rescued the deficit in active avoidance in 22q11DS mice (Figure 3E). In the active avoidance task, *Df(16)1*/+ mice injected with *Drd2* shRNA into the MGm performed significantly better than did *Df(16)1*/+ mice injected with control shRNA and similar to WT mice injected with control or *Drd2* shRNAs (Figure 3E). However, *Drd2* shRNA did not change locomotor activity between the genotypes injected with control or *Drd2* shRNAs (Figure 3F).

Synaptic transmission at thalamo-LA projections was suppressed in *Df(16)1*/+ mice injected with control shRNA into the MGm compared to that in WT mice injected with control shRNA (Figure 3G). This synaptic deficit was also rescued by injecting *Drd2* shRNA. The synaptic strength at thalamo-LA synapses of *Df(16)1*/+ mice injected with *Drd2* shRNAs was similar to that of WT mice injected with control or Drd2 shRNAs (Figure 3G). The *Df(16)1*/+ deficit in PPD at thalamo-LA synapses was also rescued by injecting Drd2 shRNA. Compared with WT controls, *Df(16)1*/+ mice injected with control shRNA showed a deficit in PPD at all interpulse intervals (Figure 3H). In contrast, PPD of thalamo-LA synapses in *Df(16)1*/+ mice injected with *Drd2* shRNA into the MGm was similar to that of WT mice injected with *Drd2* or control shRNAs (Figure 3H).

Our previous study indicated that *Dgcr8* haploinsufficiency in 22q11DS mice leads to increased *Drd2* levels in the auditory thalamus via depletion of the thalamus-enriched microRNA *miR-338* (Chun et al., 2017). To test whether *miR-338* yields emotional memory phenotypes of 22q11DS mice, we compared the behavioral performance of WT, *miR338*$^{+/-}$, and *miR338*$^{-/-}$ littermates in the fear conditioning and active avoidance tasks. Neither *miR338*$^{+/-}$ nor *miR338*$^{-/-}$ mice were deficient in fear conditioning, compared with WT littermates (Figure S7A). However, *miR338*$^{-/-}$ mice were deficient in active avoidance (Figure S7B).

Drd2 Overexpression in the Thalamus Mimics Fear Memory and Thalamo-LA Synaptic Deficits of 22q11DS Mice

To test whether abnormal elevation of Drd2s in the auditory thalamus is sufficient to cause behavioral and synaptic deficits in 22q11DS mice, we infected auditory thalamic neurons of WT mice with recombinant adeno-associated viruses (AAVs) encoding *Drd2* and GFP (*AAV-Drd2-GFP*). *AAV-GFP* was used as a control (Figure 4A). In vivo injection of *AAV-Drd2-GFP* resulted in a robust expression of GFP in neurons of the auditory thalamus, including the MGm (Figure 4B). A qRT-PCR analysis showed higher levels of the *Drd2* transcript in the auditory thalamus of WT mice injected with *AAV-Drd2-GFP* than in WT mice injected with *AAV-GFP* (Figure 4C). *Drd2* transcript levels remained unchanged in the amygdala of WT mice injected with *AAV-Drd2-GFP* or *AAV-GFP*. In the fear conditioning task, the performance of mice injected with *AAV-Drd2-GFP* was substantially poorer than that of mice injected with *AAV-GFP*, but their freezing in the pre-CS session was comparable (Figure 4D). In the active avoidance task, the performance of mice injected with *AAV-Drd2-GFP* was deficient compared to that of mice injected with *AAV-GFP* (Figure 4E), but spontaneous crossing between compartments was normal for both groups (Figure 4F).

Synaptic transmission and PPD at all interpulse intervals at thalamo-LA projections were substantially more impaired in WT mice injected with *AAV-Drd2-GFP* than in WT mice injected with *AAV-GFP* into the auditory thalamus (Figures 4G and 4H; Table S1).

Drd2-Specific Inhibitor Rescues the Thalamo-LA Deficit in Synaptic Transmission

To test the sensitivity of *Df(16)1*/+ and WT mice to Drd2 inhibitors, we measured thalamo-LA EPSCs every 30 s before and after bath application of the Drd2 inhibitor L-741,626. EPSCs evoked by the thalamic input stimulation in *Df(16)1*/+ mice were smaller than those in WT mice (Figure 5A). In a typical experiment, L-741,626 application had no noticeable effect on thalamo-LA EPSCs in WT mice, but it substantially increased thalamo-LA EPSCs in *Df(16)1*/+ mice. However, access resistance between the patch pipette and the recorded cell did not change in either genotype before or after L-741,626 application (Figure 5A). When normalized to the baseline before drug application, only thalamo-LA EPSCs of *Df(16)1*/+ mice were sensitive to L-741,626 (Figure 5B).

Synaptic Deficit at Thalamo-LA Projections in 22q11DS Mice Is Due to Reduced Probability of Glutamate Release

To identify specific mechanisms that underlie the observed synaptic deficits, we measured calcium transients at dendritic

(H) PPR at thalamo-LA projections of the following groups are shown: WT injected with control shRNA (12 neurons, three mice), *Df(16)1*/+ injected with control shRNA (15 neurons, three mice), WT injected with *Drd2* shRNA (15 neurons, three mice), and *Df(16)1*/+ injected with *Drd2* shRNA (10 neurons, three mice). Two-way repeated-measures ANOVA: $F_{3,4} = 20.75$, $p < 0.001$. Post hoc: *$p < 0.001$. Insets show representative pairs of thalamo-LA EPSCs. Scale bar, 100 pA, 50 ms. ns, not significant. Data are represented as mean ± SEM.
See also Figure S7.

OPEN
ACCESS
CellPress

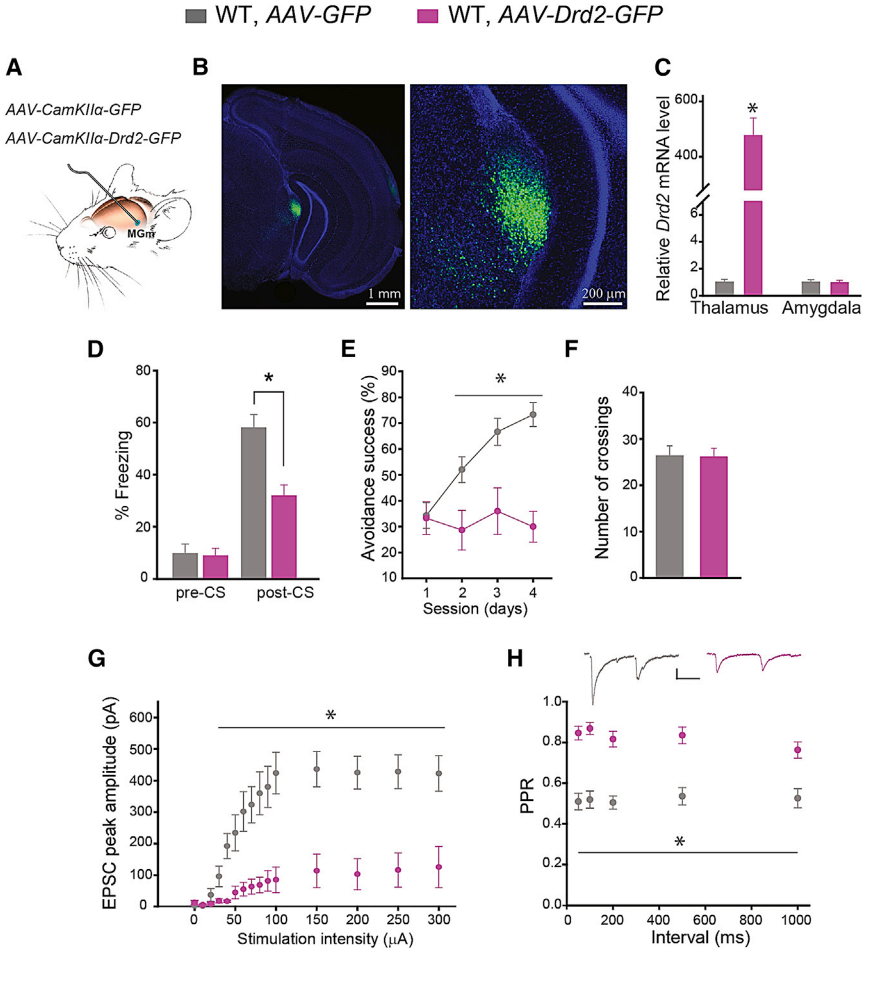

Figure 4. *Drd2* Overexpression in Excitatory Neurons of the Auditory Thalamus Mimics Fear Memory and Thalamo-LA Synaptic Deficits of 22q11DS Mice

(A) Tissue-specific Drd2 overexpression in thalamic excitatory neurons was achieved by in vivo injection of AAV expressing *AAV-Drd2-GFP* under control of the *CamKIIα* promoter (*AAV-CamKIIa-Drd2-GFP*) into the auditory thalamus. Mice injected with *AAV-GFP (AAV-CamKIIa-GFP)* were used as controls.

(B) Representative coronal sections from WT mice injected with *Drd2-GFP OE* at low (left) and high (right) magnifications. Sections were counterstained with DAPI (nuclei).

(C) Relative *Drd2* mRNA levels normalized to the respective *U6* levels in the auditory thalamus and amygdala of WT mice injected with *GFP OE* (ten mice) and WT mice injected with *Drd2-GFP OE* (ten mice). Thalamus: Mann-Whitney U test, U = 0, *p < 0.001. Amygdala: two-tailed t test, t(18) = 0.18, p = 0.86.

(D) Freezing behavior in WT mice injected with *GFP OE* or *Drd2-GFP OE* (nine mice per group). Pre-CS: Mann-Whitney U test, U = 39, p = 0.93; post-CS: two-tailed Student's t test, t(16) = 3.899, *p = 0.0013.

(E) Active avoidance performance in WT mice injected with *GFP OE* or *Drd2-GFP OE* (15 mice per group). Two-way repeated-measures ANOVA: F(1, 3) = 14.267, *p < 0.001.

(F) Total number of crossings between compartments in WT mice injected with *GFP OE* or *Drd2-GFP OE* (15 mice per group). Two-tailed Student's t test: t(28) = 0.0699, p = 0.945.

(G) Mean EPSC peak amplitude at thalamo-LA synapses of the following groups are shown: WT injected with *GFP OE* (five neurons, three mice) and WT injected with *Drd2-GFP OE* (five neurons, three mice). Two-way repeated-measures ANOVA: F(1, 14) = 18.16, *p = 0.003.

(H) PPR at different interpulse intervals measured at thalamo-LA projections of the following groups are shown: WT injected with *GFP OE* (five neurons) and WT injected with *Drd2-GFP OE* (five neurons). Two-way repeated-measures ANOVA: F(1, 4) = 60.14, p < 0.001. Insets show representative pairs of thalamo-LA EPSCs. Scale bars, 50 pA, 50 ms. Data are represented as mean ± SEM.

spines of LA neurons by two-photon calcium imaging (Figure 6A). This optical approach allows the measurement of a postsynaptic function (peak amplitude) and the presynaptic probability of release (probability of successes) of calcium transients at individual synapses (Emptage et al., 2003). Some dendritic spines responded to thalamic stimulation with calcium transients (Figures 6B and 6C). There were no differences in peak amplitudes of calcium transients in thalamic inputs to LA neurons between the genotypes (Figures 6D–6F). L-741,626 application had no effect on peak amplitudes of calcium transients (Figures 6D–6F). The probability of calcium transients in thalamic inputs to LA neurons was substantially lower in *Df(16)1/+* mice than in WT littermates (Figure 6G). This deficit was rescued by L-741,626 application (Figure 6G), suggesting that the presynaptic deficit in the probability of glutamate release at 22q11DS thalamo-LA synapses is due to elevated levels of Drd2 in the thalamus.

Chemogenetic Disruption of Synaptic Transmission at Thalamo-LA Projections Is Sufficient to Impair Fear Memory

We used a chemogenetic approach based on designer receptors exclusively activated by designer drugs (DREADDs) (Sternson and Roth, 2014) to disrupt synaptic transmission only at thalamo-LA projections. An AAV expressing the inhibitory receptor hM4Di (*AAV-hSyn-DIO-hM4Di-mCherry* [or hM4Di]) was injected into the MGm of the thalamus. To ensure that only thalamo-LA projections express hM4Di, we used the retro-DREADD approach (Roth, 2016). The canine adenovirus CAV2 encoding Cre (*CAV2-Cre*) was injected into the LA. Thus, a combination of these two viruses injected into presynaptic (thalamic) and postsynaptic (LA) sites led to the expression of hM4Di at only thalamo-LA projections, thereby reducing synaptic transmission at only these projections after hM4Di receptors interacted with the DREADD ligand clozapine-N-oxide (CNO) (Figure 7A). Three weeks after viral injections, $mCherry^+$ neurons

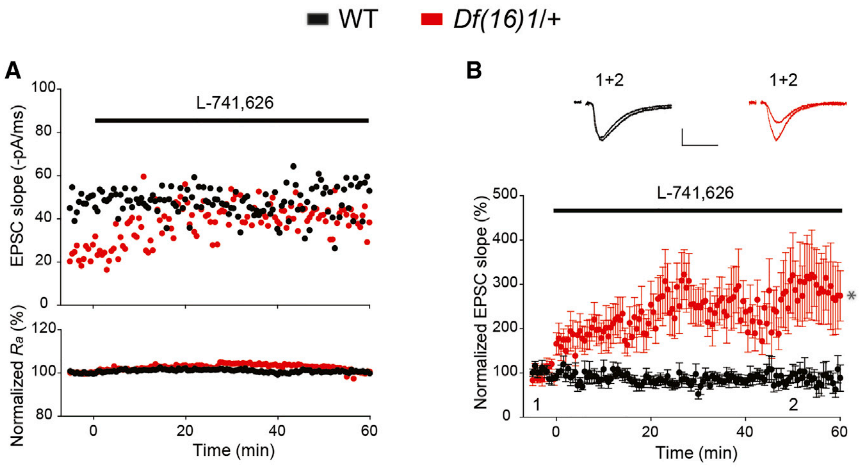

Figure 5. Drd2-Specific Inhibitor Rescues the Synaptic Transmission Deficit at Thalamo-LA Projections

(A) Representative thalamo-LA EPSC slope (top) or access resistance (R_a; bottom) before and after application of L-741,626 (20 nM) in WT and *Df(16)1/+* mice. Stimulation intensity was 60 μA for both genotypes.

(B) Mean normalized to baseline thalamo-LA EPSCs as a function of time before and after application of L-741,626 in slices from WT (n = 10) and *Df(16)1/+* (n = 10) mice. Two-tailed t test: t_{18} = 4.302, *p = 0.0004. The range of stimulation intensities was 50–100 μA for both genotypes. Insets show representative EPSC traces before (1) and after (2) drug application. Scale bar, 20 pA, 20 ms. Data are represented as mean ± SEM.

were observed in the MGm (Figure 7B), indicating that *CAV2-Cre* successfully infected axonal terminals of thalamo-LA projections in the LA. Furthermore, the *CAV2-Cre* injected into the LA retrogradely targeted MGm neurons in Ai14 reporter mice (Figure S8). The *CAV2-Cre*;*hM4Di* mice that received CNO performed more poorly in the fear conditioning task than mice that received CNO but were not injected with viruses and those *CAV2-Cre*;*hM4Di* mice that did not receive CNO (Figure 7C; Table S1). Pre-CS freezing levels were not different among the three groups. The *CAV2-Cre*;*hM4Di* mice injected with CNO also performed worse than the two control groups in the active avoidance task (Figure 7D; Table S1), but the number of crossings between the compartments was comparable (Figure 7E).

Thalamo-LA EPSCs were substantially lower in slices from *CAV2-Cre*;*hM4Di* mice treated with CNO than in slices from control mice at almost all stimulation intensities (Figure 7F; Table S1). PPR at all interpulse intervals was also lower in *CAV2-Cre*;*hM4Di* mice treated with CNO than in both groups of control mice (Figure 7G; Table S1).

DISCUSSION

Patients with 22q11DS are at increased risk (approximately 30-fold) of SCZ (Bassett and Chow, 1999; Gothelf et al., 1999; International Schizophrenia Consortium, 2008; Murphy et al., 1999; Murphy, 2002; Pulver et al., 1994; Shprintzen et al., 1992). Although the genetic lesion causing 22q11DS is well defined, the etiology of SCZ still remains unclear. Thus, studying 22q11DS holds promise in understanding the mechanisms that are common to both diseases.

The abnormal connectivity between the auditory thalamus and cortex in 22q11DS was recently described (Chun et al., 2014). This deficit is caused by the haploinsufficiency of *Dgcr8* that depletes levels of the thalamus-enriched microRNA *miR-338-3p*, which negatively regulates Drd2 in the auditory thalamus (Chun et al., 2017). The auditory thalamus includes the MGm (Winer et al., 1999) that, together with the posterior intralaminar nucleus, provides the vast majority of thalamic inputs to the amygdala (Doron and Ledoux, 1999; Russchen, 1982). Our present work indicates that the Dgcr8–Drd2 mechanism is also in play in thalamic neurons that send projections to the amygdala, specifically to the LA. The abnormal increase in Drd2 in the auditory thalamus of 22q11DS mice enhances dopamine-Drd2 signaling in thalamic projections and disrupts glutamatergic synaptic transmission at thalamocortical (TC) (Chun et al., 2014) and thalamo-LA projections. Similar to TC deficits seen in 22q11DS mice, deficits in synaptic transmission at thalamo-LA projections were caused by decreased probability of glutamate release, as revealed by reduced probability of evoked calcium transients in individual dendritic spines of LA neurons. However, in contrast to TC deficits, thalamo-LA–related behaviors are not solely dependent on *miR-338*. In *miR-338* KO mice, active avoidance behavior, but not fear conditioning behavior, was impaired, suggesting that, in addition to *miR-338*, other microRNAs mediate the Dgcr8–Drd2 mechanism in fear memory circuits of 22q11DS. Identifying these additional mediators could be an interesting direction for future research.

The findings that thalamo-LA and TC projections are impaired in 22q11DS mice (Chun et al., 2014) may lead to a general notion that synaptic transmission at all projections emanating from the thalamus are compromised in 22q11DS. This may have significant ramifications: thalamic afferents project to multiple brain areas that control different behaviors; thus, synaptic malfunction at thalamic projections may give rise to a constellation of positive, negative, and cognitive symptoms. Furthermore, this process might be controlled by a presynaptic deficiency in one brain region, the thalamus, or certain thalamic subdivisions (e.g., the auditory thalamus).

Thalamo-LA LTP is thought to underlie cue-reward learning, which may mediate goal-directed or motivational behavior (Tye et al., 2008). Although motivational behavior is thought to be encoded by neuronal circuits in several brain regions (e.g., striatum) (Drew et al., 2007; Simpson et al., 2012), the LA is considered a brain region where association between a cue and a reward is formed and where a cue is assigned motivational significance that guides reward-seeking behavior (Tye and Janak, 2007). Because both thalamo-LA synaptic transmission and LTP are significantly reduced in 22q11DS mice, it is difficult to pinpoint whether the LTP deficit is an independent phenomenon or a consequence of the deficit in synaptic transmission at these

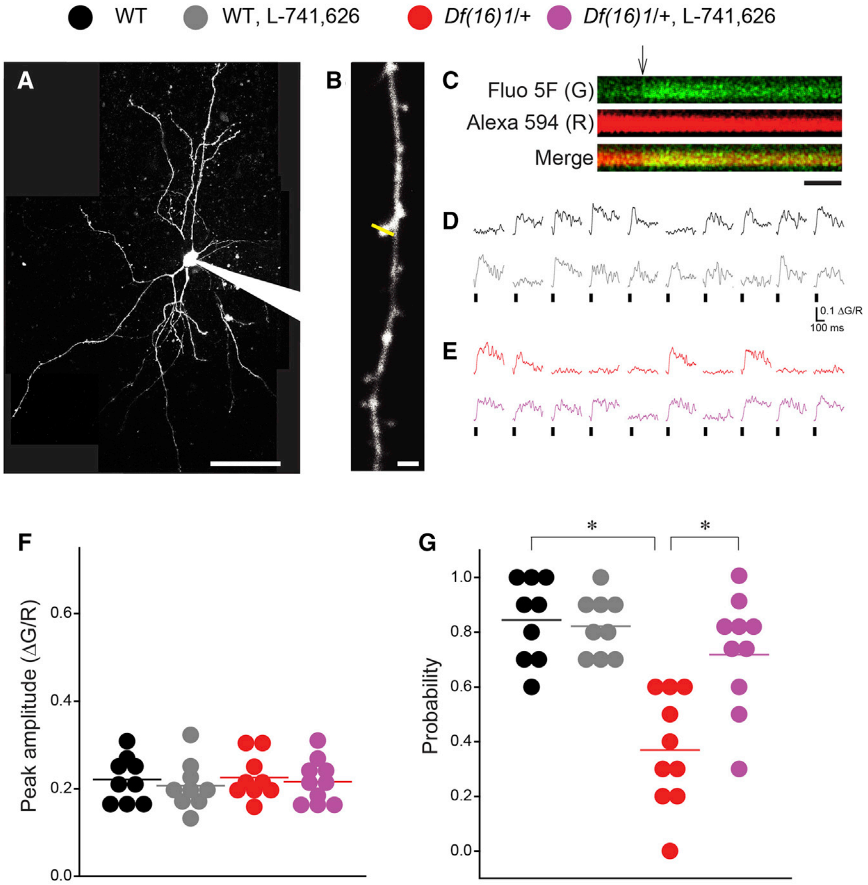

Figure 6. Reduced Probability of Glutamate Release Is Rescued by Drd2 Antagonist at Thalamic Inputs to LA in 22q11DS Mice

(A) Image of an LA neuron filled with the Alexa Fluor 594 and Fluo-5F through the patch pipette. Scale bar, 50 μm.

(B) Image of a dendrite with dendritic spines in an LA neuron. The line shows the direction of a line scan. Scale bar, 2 μm.

(C) Representative fluorescence images of a line scan through an individual dendritic spine of an LA neuron. The green channel (G) shows Fluo-5F fluorescence. The red channel (R) shows Alexa Fluor 594 fluorescence. Scale bar, 50 ms.

(D and E) Examples of line scans as a function of time in the same dendritic spines of WT (D) and *Df(16)1/+* (E) mice before and after application of the Drd2 antagonist L-741,626.

(F and G) Mean peak amplitudes (F) and probabilities (G) of calcium transients in dendritic spines, which are the sites of thalamic inputs to the LA in WT (nine spines, six animals) and *Df(16)1/+* (ten spines, six animals) mice before and after the application of L-741,626. Two-way repeated-measures ANOVA: peak amplitude: $F_{1,17} = 0.031$, $p = 0.862$. Probability: $F_{1,17} = 20.442$, $^{*}p < 0.001$. Post hoc: WT, before versus after L-741,626, $p = 0.754$; *Df(16)1/+*, before versus after L-741,626, $^{*}p < 0.001$; WT (no L-741,626) versus *Df(16)1/+* (no L-741,626), $^{*}p < 0.001$; WT (with L-741,626) versus *Df(16)1/+* (with L-741,626), $p = 0.201$.

projections. Our experiments showed that disruption of synaptic transmission at thalamo-LA projections is sufficient to diminish performance in fear conditioning and active avoidance. Whether an impaired LTP at these projections is an intermediary for these behavioral phenotypes will require further investigation.

From a clinical standpoint, emotional memory deficits in 22q11DS mice could be related to the negative symptoms of SCZ. Past studies have noted a relation between emotional memory deficits and negative symptoms (Hall et al., 2007; Horan et al., 2006; Olsen et al., 2015). Individuals with SCZ have normal emotional responses to positive stimuli at the moment of exposure or consumption, but they do not use positive experiences to guide their decisions to engage in the same or similar activities in the future (Gard et al., 2007; Herbener, 2008; Horan et al., 2006). These impairments in memory of emotional experiences, particularly over longer periods of time, are believed to contribute to goal-directed behaviors and negative symptoms such as amotivation and anhedonia (Herbener, 2008). Negative symptoms can be alleviated by typical or first-generation antipsychotics (Miyamoto et al., 2002, 2005, 2012), which is consistent with our findings that thalamo-LA and emotional memory deficits in 22q11DS mice were rescued by Drd2 antagonists or thalamic Drd2 small interfering RNAs. However, note that antipsychotics are less effective treatment for negative symptoms than for positive symptoms (Leucht et al., 2009).

In summary, we showed that mouse models of 22q11DS exhibit a specific disruption of synaptic transmission at thalamo-LA projections, which leads to deficits in emotional memory that might underlie some negative symptoms associated with SCZ. This synaptic deficit is caused by haploinsufficiency of the microRNA-processing gene *Dgcr8* and is mediated by abnormal elevation of *Drd2*s in the auditory thalamus. In our view, this Dgcr8–Drd2 mechanism at thalamo-LA projections may underlie some pathogenic mechanisms related to emotional memory disturbances in 22q11DS and associated cases of psychiatric disease.

EXPERIMENTAL PROCEDURES

Animals

Both male and female mice (4–5 months old) were used for all experiments. The generation of *Df(16)1/+*, *Dgcr8* floxed, and *miR338*$^{-/-}$ mouse lines has been reported previously (Chun et al., 2017; Lindsay et al., 1999; Yi et al., 2009). *Gbx2*^{CreER} (JAX stock no. 22135) and Ai14 (JAX stock no. 007914) mouse strains were purchased from the Jackson Laboratory. For most experiments, the experimenters were blinded to the genotype or treatment. The care and use of animals were reviewed and approved by the Institutional Animal Care and Use Committee at St. Jude Children's Research Hospital.

Behavioral Analyses

Fear Conditioning Test

To test auditory cued fear conditioning, a mouse was placed in a conditioning chamber with the white house light on and allowed to explore the testing chamber for 2 min before a discrete CS was delivered in the form of a tone

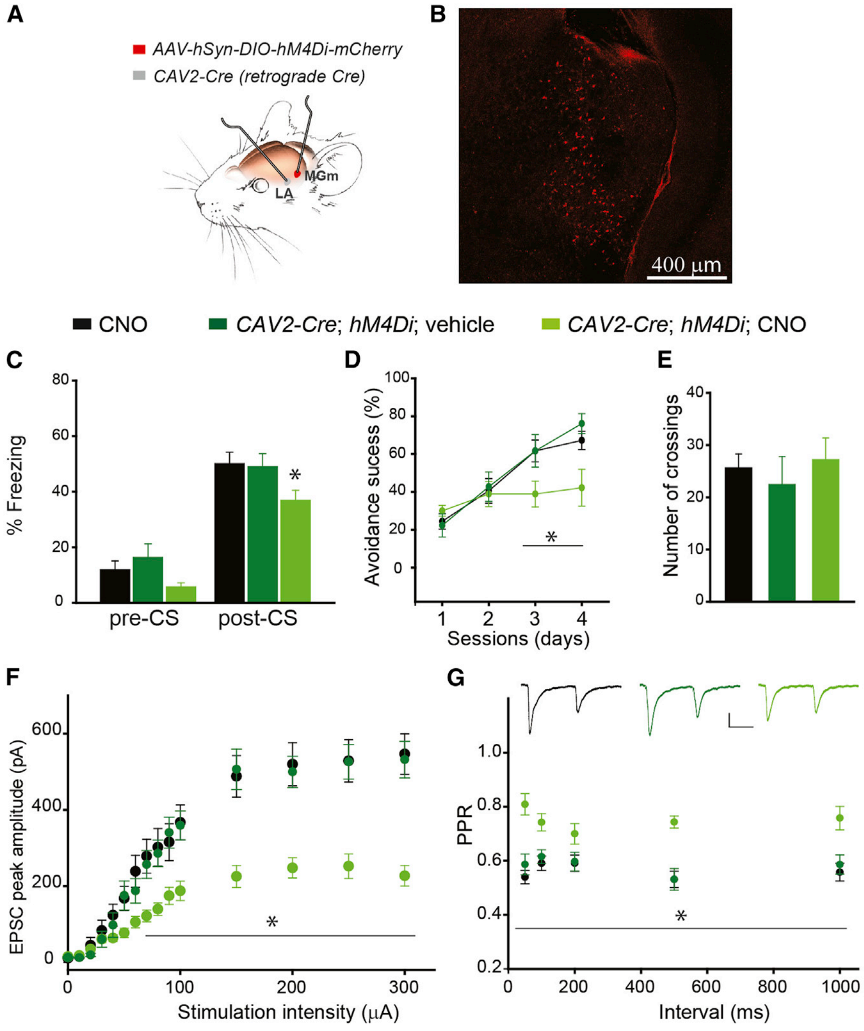

Figure 7. Specific Synaptic Silencing of Thalamo-LA Projections Is Sufficient to Impair Associative Fear Memory

(A) Schematics of the retro-DREADD approach to silence the thalamo-LA projections. MGm neurons were infected with Cre-dependent *AAV-hSyn-DIO-hM4Di-mCherry,* and LA neurons were infected with *CAV2-Cre*.

(B) Representative fluorescent image of the auditory thalamus in *CAV2-Cre*;*hM4Di* mice injected with CNO.

(C) Fear conditioning in CNO-administered WT mice (CNO, 16 animals), vehicle-administered *CAV2-Cre*;*hM4Di* mice (*CAV2-Cre*;*hM4Di*;vehicle, 14 animals), and CNO-administered *CAV2-Cre*;*hM4Di* mice (*CAV2-Cre*;*hM4Di*, CNO, 16 animals). Pre-CS: Kruskal-Wallis one-way ANOVA on ranks: H_2 = 3.784, p = 0.151. Post-CS: one-way ANOVA: F_2 = 3.252, *p = 0.048. Post hoc: *CAV2-Cre*;*hM4Di*, CNO versus CNO: *p = 0.05; CNO versus *CAV2-Cre*;*hM4Di*;vehicle: p = 0.865.

(D) Active avoidance in CNO, *CAV2-Cre*;*hM4Di*; vehicle, and *CAV2-Cre*;*hM4Di*, CNO mice (nine mice per group). Two-way ANOVA: F(1, 3) = 4.032, *p = 0.012. Post hoc, *CAV2-Cre*;*hM4Di*, CNO versus CNO: day 3, p = 0.014; day 4, p = 0.006.

(E) Mean total number of crossings between compartments in CNO, *CAV2-Cre*;*hM4Di*;vehicle, and *CAV2-Cre*;*hM4Di*, CNO mice (nine mice per group). One-way ANOVA: F_2 = 0.338, p = 0.717.

(F) Thalamo-LA EPSCs as a function of stimulation intensity in slices from CNO (11 neurons, four mice), *CAV2-Cre*;*hM4Di*;vehicle (10 neurons, four mice), and *CAV2-Cre*;*hM4Di*, CNO mice (12 neurons, three mice). Two-way repeated-measures ANOVA: F(2, 14) = 11.71, p < 0.001. Post hoc: *p < 0.01, except for *CAV2-Cre*;*hM4Di*;vehicle versus CNO, p = 0.723.

(G) PPR at thalamo-LA projections from CNO (15 neurons, five mice), *CAV2-Cre*;*hM4Di*;vehicle (14 neurons, four mice), and *CAV2-Cre*;*hM4Di*, CNO mice (13 neurons, three mice). Two-way repeated-measures ANOVA: F(2, 4) = 17.29, p < 0.001. Post hoc: *p < 0.01, except for *CAV2-Cre*;*hM4Di*;vehicle versus CNO, p = 0.624. Insets, representative traces of pairs of thalamo-LA EPSCs. Scale bar, 50 pA, 50 ms. Data are represented as mean ± SEM.

See also Figure S8.

(30 s, 10 kHz, 75-dB sound pressure level [SPL]). Within the last 2 s of the tone, a US was delivered in the form of a mild footshock (0.5 mA, 2 s). Mice were allowed to recover for 1 min, and then another three CS-US pairs were delivered. After the last CS-US pairing, mice remained in the conditioning chamber for 1 min and were then returned to the home cage. Approximately 1 or 24 hr later, mice were placed in a new environment with the light off and allowed to explore for 2 min, followed by exposure to only the CS tone for 30 s. After a recovery period of 30 s, the tone exposure and recovery period steps were repeated three times. The percentages of freezing times in the training period and during the pre-CS and post-CS periods on the test day were compared across groups using Video Freeze software (Med Associates).

Active Avoidance Test

On the first day, mice were habituated in a chamber for 5 min. The total number of spontaneous crossings between compartments was recorded. On the second day, mice were given auditory cued fear conditioning training. Mice were placed in compartment A (unsafe compartment) with the gate closed, and a CS (10 kHz, 75 dB SPL, 5 s) was given while the gate between compartment A and compartment B (safe compartment) was open. Mice that did not cross to compartment B after 5 s of CS delivery received a US (mild electric shock, 0.4 mA) for 25 s or until they crossed to compartment B. The escape success was measured as the percentage of entries into compartment B during the CS presentation. Each mouse was given 20 CS-US pairs each day (randomized intertrial interval) for 4 consecutive days.

Whole-Cell Recordings

Coronal brain slices (300 μm thick) containing the amygdala were prepared as previously described (Cho et al., 2011). Briefly, mouse brains were quickly removed and placed in 4°C dissecting artificial cerebrospinal fluid (ACSF) containing 125 mM choline Cl, 2.5 mM KCl, 0.4 mM $CaCl_2$, 6 mM $MgCl_2$, 1.25 mM NaH_2PO_4, 26 mM $NaHCO_3$, and 20 mM glucose (300–310 mOsm), with 95% O_2/5% CO_2. After a 1-hr incubation in ACSF (125 mM NaCl, 2.5 mM KCl, 2 mM $CaCl_2$, 2 mM $MgCl_2$, 1.25 mM NaH_2PO_4, 26 mM $NaHCO_3$, and 20 mM glucose [300–310 mOsm], with 95% O_2/5% CO_2) at room temperature, slices were transferred to the recording chamber and superfused (2–3 mL/min) with 30°C–32°C ACSF. Whole-cell recordings of EPSCs were obtained from principal neurons in the LA under visual guidance (Dodt gradient contrast and two-photon imaging) with a Multiclamp 700B amplifier and pCLAMP 10.0 software (Molecular Devices). Synaptic responses were evoked by stimulating the fibers in the external capsule (cortical input) or the internal capsule (thalamic input). Under our experimental conditions, thalamic and cortical inputs

converging on the same LA neurons were stimulated independently, because the sum of thalamo-LA EPSCs and cortico-LA EPSCs, when they were triggered individually, was nearly identical to that of the EPSCs when both inputs were simultaneously stimulated (data not shown). The independence of inputs was further confirmed by the observation that stimulation of the cortical input did not affect the thalamo-LA EPSC (evoked with a 50-ms delay), and that of the thalamic input did not affect the cortico-LA EPSC (evoked with a 50-ms delay), which was consistent with the previous results (Cho et al., 2011).

Patch electrodes (3- to 5-MΩ resistance) contained the following internal solution: 125 mM cesium methanesulfonate, 2 mM CsCl, 10 mM HEPES, 0.1 mM EGTA, 4 mM MgATP, 0.3 mM NaGTP, 5 mM tetraethylammonium, 10 mM Na_2 creatine phosphate, 5 mM QX-314 (adjusted to pH 7.4 with CsOH [290–295 mOsm]). Synaptic responses were filtered at 5 kHz and digitized at 20 kHz. To evoke synaptic responses, square current pulses (100-μs duration of various intensities) were applied through a thin tungsten electrode. Membrane potential was held constant at −70 mV throughout the experiments in the voltage-clamp mode.

Current-clamp recordings were conducted using the following internal solution: 115 mM potassium gluconate; 20 mM KCl; 10 mM HEPES; 4 mM $MgCl_2 \cdot 6H_2O$; 0.1 mM EGTA; 4 mM Na_2ATP; 0.4 mM NaGTP; 10 mM Na_2 creatine phosphate; and 30 μM Alexa Fluor 594, pH 7.3–7.4 (290–295 mOsm). LTP at cortico-LA and thalamo-LA inputs was recorded in the voltage-clamp mode. LTP at the cortico-LA input was induced by 80 presynaptic pulses delivered at 2 Hz. An LA neuron was held at +30 mV for the duration of presynaptic stimulation. Postsynaptically and presynaptically expressed LTPs at the thalamo-LA input were induced by 240 paired presynaptic stimuli (50-ms interpulse interval) delivered at 2 Hz to the presynaptic fibers. An LA neuron was held at +30 mV or −70 mV to express LTP post- or pre-synaptically, respectively (Shin et al., 2010). In LTP experiments, the modified ACSF (100 μM picrotoxin, 2.5 mM $CaCl_2$, 1 mM $MgCl_2$, room temperature) was used.

Two-Photon Imaging

The Ultima imaging system (Prairie Technologies), equipped with a titanium: sapphire Chameleon Ultra femtosecond-pulsed laser (Coherent) and a 60× (0.9 NA) water-immersion infrared objective (Olympus), was used. Briefly, Alexa Fluor 594 (30 μM) and Fluo-5F (300 μM) were loaded into the principal LA neurons with the internal pipette solution. Alexa Fluor 594 and Fluo-5F were excited with laser pulses at 820 nm, and changes in both red and green fluorescence were simultaneously measured in the line-scan mode (500 Hz) in spine heads when an electrical stimulation was applied to the thalamic input of the LA. To measure calcium transient amplitudes and probabilities of success, 10–20 line scans were analyzed as changes in Fluo-5F fluorescence normalized to Alexa Fluor 594 fluorescence ($\Delta G/R$).

qRT-PCR

Total RNA was isolated from brain regions (i.e., the auditory thalamus containing the MGm and amygdala) by using the mirVana microRNA Isolation Kit (Life Technologies). The iScript kit (Bio-Rad) was used to synthesize cDNA from mRNA. The qRT-PCR was performed using SYBR Green (Life Technologies), with the following primers: *Drd2* forward (5′-GGATGTCATGATGTGCACAGC-3′), *Drd2* reverse (5′-CGCTTGCGGAGAACGATG-3′), *Dgcr8* forward (5′-CCACGAC CATCCTCAGACATTG-3′), *Dgcr8* reverse (5′-ATGAAAATCTCCCCTCCCCAC AGCC-3′), *U6* forward (5′-CGCTTCGGCAGCACATATAC-3′), and *U6* reverse (5′-TTCACGAATTTGCGTGTCAT-3′). Expression levels of *Drd2* were normalized to the housekeeping gene *U6* for each sample. Samples for each mouse were run in triplicate.

Statistical Analyses

All statistical data were computed using the Sigma Plot 12.5 software. Parametric or nonparametric tests were chosen based on the normality and variance of data distribution. Independent or paired two-tailed t tests, a Mann-Whitney rank-sum U test, a one-way ANOVA/Kruskal-Wallis one-way ANOVA on ranks H test followed by a multiple comparison procedure (Dunn's method), and a two-way ANOVA/two-way repeated-measures ANOVA with one-factor repetition followed by a Holm-Sidak multiple comparison procedure were used. F values were reported for ANOVA. $p < 0.05$ was considered significant.

SUPPLEMENTAL INFORMATION

Supplemental Information includes Supplemental Experimental Procedures, eight figures, and one table and can be found with this article online at http://dx.doi.org/10.1016/j.celrep.2017.05.002.

AUTHOR CONTRIBUTIONS

T.-Y.E. and S.S.Z. designed the study. T.-Y.E. performed behavioral, immunohistochemical, and molecular experiments. I.T.B. performed electrophysiological and two-photon imaging experiments. J.Y. assisted with qRT-PCR experiments. K.A. assisted with molecular and immunohistochemistry experiments. S.S.Z. provided reagents and equipment. S.S.Z. and T.-Y.E. wrote the manuscript.

ACKNOWLEDGMENTS

We thank Martin Darvas of the University of Washington for the generous gift of Cav2-Cre viruses, members of the Zakharenko lab for providing comments, and Vani Shanker and Angela McArthur for editing the manuscript. This work was supported by NIH grants MH097742 and MH095810, the NARSAD Independent Investigator Award, and ALSAC (to S.S.Z.) and by the Garwood postdoctoral fellowship (to T.-Y.E.). The funding sources had no role in the study design, data collection, data analysis, decision to publish, or preparation of the manuscript.

Received: February 1, 2017
Revised: March 24, 2017
Accepted: April 27, 2017
Published: May 23, 2017

REFERENCES

Azuma, S., Yamamoto, T., and Kawamura, Y. (1984). Studies on gustatory responses of amygdaloid neurons in rats. Exp. Brain Res. *56*, 12–22.

Bartel, D.P. (2009). MicroRNAs: target recognition and regulatory functions. Cell *136*, 215–233.

Bassett, A.S., and Chow, E.W. (1999). 22q11 deletion syndrome: a genetic subtype of schizophrenia. Biol. Psychiatry *46*, 882–891.

Bassett, A.S., McDonald-McGinn, D.M., Devriendt, K., Digilio, M.C., Goldenberg, P., Habel, A., Marino, B., Oskarsdottir, S., Philip, N., Sullivan, K., et al.; International 22q11.2 Deletion Syndrome Consortium (2011). Practical guidelines for managing patients with 22q11.2 deletion syndrome. J. Pediatr. *159*, 332–339.e1.

Bayazitov, I.T., Westmoreland, J.J., and Zakharenko, S.S. (2013). Forward suppression in the auditory cortex is caused by the Ca(v)3.1 calcium channel-mediated switch from bursting to tonic firing at thalamocortical projections. J. Neurosci. *33*, 18940–18950.

Blundon, J.A., Bayazitov, I.T., and Zakharenko, S.S. (2011). Presynaptic gating of postsynaptically expressed plasticity at mature thalamocortical synapses. J. Neurosci. *31*, 16012–16025.

Cain, C.K., and LeDoux, J.E. (2008). Brain mechanisms of Pavlovian and instrumental aversive conditioning. In Handbook of Anxiety and Fear, R.J. Blanchard, D.C. Blanchard, G. Griebel, and D.J. Nutt, eds. (Elsevier), pp. 103–124.

Campbell, L.E., Daly, E., Toal, F., Stevens, A., Azuma, R., Catani, M., Ng, V., van Amelsvoort, T., Chitnis, X., Cutter, W., et al. (2006). Brain and behaviour in children with 22q11.2 deletion syndrome: a volumetric and voxel-based morphometry MRI study. Brain *129*, 1218–1228.

Campbell, L., McCabe, K., Leadbeater, K., Schall, U., Loughland, C., and Rich, D. (2010). Visual scanning of faces in 22q11.2 deletion syndrome: Attention to the mouth or the eyes? Psychiatry Res. *177*, 211–215.

Chatterjee, M., Li, K., Chen, L., Maisano, X., Guo, Q., Gan, L., and Li, J.Y. (2012). Gbx2 regulates thalamocortical axon guidance by modifying the LIM and Robo codes. Development *139*, 4633–4643.

Chen, L., Guo, Q., and Li, J.Y. (2009). Transcription factor Gbx2 acts cell-non-autonomously to regulate the formation of lineage-restriction boundaries of the thalamus. Development *136*, 1317–1326.

Cho, J.H., Bayazitov, I.T., Meloni, E.G., Myers, K.M., Carlezon, W.A., Jr., Zakharenko, S.S., and Bolshakov, V.Y. (2011). Coactivation of thalamic and cortical pathways induces input timing-dependent plasticity in amygdala. Nat. Neurosci. *15*, 113–122.

Chow, E.W., Watson, M., Young, D.A., and Bassett, A.S. (2006). Neurocognitive profile in 22q11 deletion syndrome and schizophrenia. Schizophr. Res. *87*, 270–278.

Chun, S., Bayazitov, I.T., Blundon, J.A., and Zakharenko, S.S. (2013). Thalamocortical long-term potentiation becomes gated after the early critical period in the auditory cortex. J. Neurosci. *33*, 7345–7357.

Chun, S., Westmoreland, J.J., Bayazitov, I.T., Eddins, D., Pani, A.K., Smeyne, R.J., Yu, J., Blundon, J.A., and Zakharenko, S.S. (2014). Specific disruption of thalamic inputs to the auditory cortex in schizophrenia models. Science *344*, 1178–1182.

Chun, S., Du, F., Westmoreland, J.J., Han, S.B., Wang, Y.D., Eddins, D., Bayazitov, I.T., Devaraju, P., Yu, J., Mellado Lagarde, M.M., et al. (2017). Thalamic miR-338-3p mediates auditory thalamocortical disruption and its late onset in models of 22q11.2 microdeletion. Nat. Med. *23*, 39–48.

Doron, N.N., and Ledoux, J.E. (1999). Organization of projections to the lateral amygdala from auditory and visual areas of the thalamus in the rat. J. Comp. Neurol. *412*, 383–409.

Drew, M.R., Simpson, E.H., Kellendonk, C., Herzberg, W.G., Lipatova, O., Fairhurst, S., Kandel, E.R., Malapani, C., and Balsam, P.D. (2007). Transient overexpression of striatal D2 receptors impairs operant motivation and interval timing. J. Neurosci. *27*, 7731–7739.

Earls, L.R., Fricke, R.G., Yu, J., Berry, R.B., Baldwin, L.T., and Zakharenko, S.S. (2012). Age-dependent microRNA control of synaptic plasticity in 22q11 deletion syndrome and schizophrenia. J. Neurosci. *32*, 14132–14144.

Emptage, N.J., Reid, C.A., Fine, A., and Bliss, T.V. (2003). Optical quantal analysis reveals a presynaptic component of LTP at hippocampal Schaffer-associational synapses. Neuron *38*, 797–804.

Engel, M., Fritzsche, A., and Lincoln, T.M. (2015). Subclinical negative symptoms and the anticipation, experience and recall of emotions related to social interactions: An experimental study. Psychiatry Res. *230*, 350–356.

Gard, D.E., Kring, A.M., Gard, M.G., Horan, W.P., and Green, M.F. (2007). Anhedonia in schizophrenia: distinctions between anticipatory and consummatory pleasure. Schizophr. Res. *93*, 253–260.

Gil, Z., Connors, B.W., and Amitai, Y. (1999). Efficacy of thalamocortical and intracortical synaptic connections: quanta, innervation, and reliability. Neuron *23*, 385–397.

Gothelf, D., Frisch, A., Munitz, H., Rockah, R., Laufer, N., Mozes, T., Hermesh, H., Weizman, A., and Frydman, M. (1999). Clinical characteristics of schizophrenia associated with velo-cardio-facial syndrome. Schizophr. Res. *35*, 105–112.

Green, T., Gothelf, D., Glaser, B., Debbane, M., Frisch, A., Kotler, M., Weizman, A., and Eliez, S. (2009). Psychiatric disorders and intellectual functioning throughout development in velocardiofacial (22q11.2 deletion) syndrome. J. Am. Acad. Child Adolesc. Psychiatry *48*, 1060–1068.

Hall, J., Harris, J.M., McKirdy, J.W., Johnstone, E.C., and Lawrie, S.M. (2007). Emotional memory in schizophrenia. Neuropsychologia *45*, 1152–1159.

Herbener, E.S. (2008). Emotional memory in schizophrenia. Schizophr. Bull. *34*, 875–887.

Herbener, E.S., Rosen, C., Khine, T., and Sweeney, J.A. (2007). Failure of positive but not negative emotional valence to enhance memory in schizophrenia. J. Abnorm. Psychol. *116*, 43–55.

Horan, W.P., Green, M.F., Kring, A.M., and Nuechterlein, K.H. (2006). Does anhedonia in schizophrenia reflect faulty memory for subjectively experienced emotions? J. Abnorm. Psychol. *115*, 496–508.

International Schizophrenia Consortium (2008). Rare chromosomal deletions and duplications increase risk of schizophrenia. Nature *455*, 237–241.

Kring, A.M., and Elis, O. (2013). Emotion deficits in people with schizophrenia. Annu. Rev. Clin. Psychol. *9*, 409–433.

Lajiness-O'Neill, R.R., Beaulieu, I., Titus, J.B., Asamoah, A., Bigler, E.D., Bawle, E.V., and Pollack, R. (2005). Memory and learning in children with 22q11.2 deletion syndrome: evidence for ventral and dorsal stream disruption? Child Neuropsychol. *11*, 55–71.

LeDoux, J.E. (2000). Emotion circuits in the brain. Annu. Rev. Neurosci. *23*, 155–184.

LeDoux, J. (2003). The emotional brain, fear, and the amygdala. Cell. Mol. Neurobiol. *23*, 727–738.

Leleu, A., Saucourt, G., Rigard, C., Chesnoy, G., Baudouin, J.Y., Rossi, M., Edery, P., Franck, N., and Demily, C. (2016). Facial emotion perception by intensity in children and adolescents with 22q11.2 deletion syndrome. Eur. Child Adolesc. Psychiatry *25*, 297–310.

Leucht, S., Corves, C., Arbter, D., Engel, R.R., Li, C., and Davis, J.M. (2009). Second-generation versus first-generation antipsychotic drugs for schizophrenia: a meta-analysis. Lancet *373*, 31–41.

Lindsay, E.A., Botta, A., Jurecic, V., Carattini-Rivera, S., Cheah, Y.C., Rosenblatt, H.M., Bradley, A., and Baldini, A. (1999). Congenital heart disease in mice deficient for the DiGeorge syndrome region. Nature *401*, 379–383.

Madisen, L., Zwingman, T.A., Sunkin, S.M., Oh, S.W., Zariwala, H.A., Gu, H., Ng, L.L., Palmiter, R.D., Hawrylycz, M.J., Jones, A.R., et al. (2010). A robust and high-throughput Cre reporting and characterization system for the whole mouse brain. Nat. Neurosci. *13*, 133–140.

Mahanty, N.K., and Sah, P. (1998). Calcium-permeable AMPA receptors mediate long-term potentiation in interneurons in the amygdala. Nature *394*, 683–687.

Maren, S., and Quirk, G.J. (2004). Neuronal signalling of fear memory. Nat. Rev. Neurosci. *5*, 844–852.

McCabe, K., Rich, D., Loughland, C.M., Schall, U., and Campbell, L.E. (2011). Visual scanpath abnormalities in 22q11.2 deletion syndrome: is this a face specific deficit? Psychiatry Res. *189*, 292–298.

McDonald-McGinn, D.M., and Sullivan, K.E. (2011). Chromosome 22q11.2 deletion syndrome (DiGeorge syndrome/velocardiofacial syndrome). Medicine (Baltimore) *90*, 1–18.

McKernan, M.G., and Shinnick-Gallagher, P. (1997). Fear conditioning induces a lasting potentiation of synaptic currents in vitro. Nature *390*, 607–611.

Miyamoto, S., Duncan, G.E., Goff, D.C., and Lieberman, J.A. (2002). Therapeutics of schizophrenia. In Neuropsychopharmacology: The Fifth Generation of Progress, K.L. Davis, D. Charney, J.T. Coyle, and C. Nemeroff, eds. (Lippincott, Williams & Wilkins), pp. 775–807.

Miyamoto, S., Duncan, G.E., Marx, C.E., and Lieberman, J.A. (2005). Treatments for schizophrenia: a critical review of pharmacology and mechanisms of action of antipsychotic drugs. Mol. Psychiatry *10*, 79–104.

Miyamoto, S., Miyake, N., Jarskog, L.F., Fleischhacker, W.W., and Lieberman, J.A. (2012). Pharmacological treatment of schizophrenia: a critical review of the pharmacology and clinical effects of current and future therapeutic agents. Mol. Psychiatry *17*, 1206–1227.

Murphy, K.C. (2002). Schizophrenia and velo-cardio-facial syndrome. Lancet *359*, 426–430.

Murphy, K.C., Jones, L.A., and Owen, M.J. (1999). High rates of schizophrenia in adults with velo-cardio-facial syndrome. Arch. Gen. Psychiatry *56*, 940–945.

Nakashima, M., Uemura, M., Yasui, K., Ozaki, H.S., Tabata, S., and Taen, A. (2000). An anterograde and retrograde tract-tracing study on the projections from the thalamic gustatory area in the rat: distribution of neurons projecting to the insular cortex and amygdaloid complex. Neurosci. Res. *36*, 297–309.

Olsen, E.K., Bjorkquist, O.A., Bodapati, A.S., Shankman, S.A., and Herbener, E.S. (2015). Associations between trait anhedonia and emotional memory deficits in females with schizophrenia versus major depression. Psychiatry Res. *230*, 323–330.

Pulver, A.E., Nestadt, G., Goldberg, R., Shprintzen, R.J., Lamacz, M., Wolyniec, P.S., Morrow, B., Karayiorgou, M., Antonarakis, S.E., Housman, D.,

et al. (1994). Psychotic illness in patients diagnosed with velo-cardio-facial syndrome and their relatives. J. Nerv. Ment. Dis. *182*, 476–478.

Quirk, G.J., Repa, C., and LeDoux, J.E. (1995). Fear conditioning enhances short-latency auditory responses of lateral amygdala neurons: parallel recordings in the freely behaving rat. Neuron *15*, 1029–1039.

Quirk, G.J., Armony, J.L., and LeDoux, J.E. (1997). Fear conditioning enhances different temporal components of tone-evoked spike trains in auditory cortex and lateral amygdala. Neuron *19*, 613–624.

Rogan, M.T., Stäubli, U.V., and LeDoux, J.E. (1997). Fear conditioning induces associative long-term potentiation in the amygdala. Nature *390*, 604–607.

Romanski, L.M., and LeDoux, J.E. (1992). Equipotentiality of thalamo-amygdala and thalamo-cortico-amygdala circuits in auditory fear conditioning. J. Neurosci. *12*, 4501–4509.

Rosenkranz, J.A., and Grace, A.A. (2002). Dopamine-mediated modulation of odour-evoked amygdala potentials during pavlovian conditioning. Nature *417*, 282–287.

Roth, B.L. (2016). DREADDs for neuroscientists. Neuron *89*, 683–694.

Rumpel, S., LeDoux, J., Zador, A., and Malinow, R. (2005). Postsynaptic receptor trafficking underlying a form of associative learning. Science *308*, 83–88.

Russchen, F.T. (1982). Amygdalopetal projections in the cat. II. Subcortical afferent connections. A study with retrograde tracing techniques. J. Comp. Neurol. *207*, 157–176.

Scambler, P.J., Kelly, D., Lindsay, E., Williamson, R., Goldberg, R., Shprintzen, R., Wilson, D.I., Goodship, J.A., Cross, I.E., and Burn, J. (1992). Velo-cardio-facial syndrome associated with chromosome 22 deletions encompassing the DiGeorge locus. Lancet *339*, 1138–1139.

Shin, R.M., Tully, K., Li, Y., Cho, J.H., Higuchi, M., Suhara, T., and Bolshakov, V.Y. (2010). Hierarchical order of coexisting pre- and postsynaptic forms of long-term potentiation at synapses in amygdala. Proc. Natl. Acad. Sci. USA *107*, 19073–19078.

Shprintzen, R.J. (2000). Velo-cardio-facial syndrome: a distinctive behavioral phenotype. Ment. Retard. Dev. Disabil. Res. Rev. *6*, 142–147.

Shprintzen, R.J., Goldberg, R., Golding-Kushner, K.J., and Marion, R.W. (1992). Late-onset psychosis in the velo-cardio-facial syndrome. Am. J. Med. Genet. *42*, 141–142.

Simpson, E.H., Waltz, J.A., Kellendonk, C., and Balsam, P.D. (2012). Schizophrenia in translation: dissecting motivation in schizophrenia and rodents. Schizophr. Bull. *38*, 1111–1117.

Stark, K.L., Xu, B., Bagchi, A., Lai, W.S., Liu, H., Hsu, R., Wan, X., Pavlidis, P., Mills, A.A., Karayiorgou, M., and Gogos, J.A. (2008). Altered brain microRNA biogenesis contributes to phenotypic deficits in a 22q11-deletion mouse model. Nat. Genet. *40*, 751–760.

Sternson, S.M., and Roth, B.L. (2014). Chemogenetic tools to interrogate brain functions. Annu. Rev. Neurosci. *37*, 387–407.

Tsvetkov, E., Carlezon, W.A., Benes, F.M., Kandel, E.R., and Bolshakov, V.Y. (2002). Fear conditioning occludes LTP-induced presynaptic enhancement of synaptic transmission in the cortical pathway to the lateral amygdala. Neuron *34*, 289–300.

Tye, K.M., and Janak, P.H. (2007). Amygdala neurons differentially encode motivation and reinforcement. J. Neurosci. *27*, 3937–3945.

Tye, K.M., Stuber, G.D., de Ridder, B., Bonci, A., and Janak, P.H. (2008). Rapid strengthening of thalamo-amygdala synapses mediates cue-reward learning. Nature *453*, 1253–1257.

Viaene, A.N., Petrof, I., and Sherman, S.M. (2011). Synaptic properties of thalamic input to the subgranular layers of primary somatosensory and auditory cortices in the mouse. J. Neurosci. *31*, 12738–12747.

Wang, Y., Medvid, R., Melton, C., Jaenisch, R., and Blelloch, R. (2007). DGCR8 is essential for microRNA biogenesis and silencing of embryonic stem cell self-renewal. Nat. Genet. *39*, 380–385.

Wilson, T.D., and Gilbert, D.T. (2003). Affective forecasting. Adv. Exp. Soc. Psychol. *35*, 345–411.

Winer, J.A., Kelly, J.B., and Larue, D.T. (1999). Neural architecture of the rat medial geniculate body. Hear. Res. *130*, 19–41.

Yi, R., Pasolli, H.A., Landthaler, M., Hafner, M., Ojo, T., Sheridan, R., Sander, C., O'Carroll, D., Stoffel, M., Tuschl, T., and Fuchs, E. (2009). DGCR8-dependent microRNA biogenesis is essential for skin development. Proc. Natl. Acad. Sci. USA *106*, 498–502.

Zucker, R.S., and Regehr, W.G. (2002). Short-term synaptic plasticity. Annu. Rev. Physiol. *64*, 355–405.

Cell Reports, Volume *19*

Supplemental Information

Schizophrenia-Related Microdeletion Impairs Emotional Memory through MicroRNA-Dependent Disruption of Thalamic Inputs to the Amygdala

Tae-Yeon Eom, Ildar T. Bayazitov, Kara Anderson, Jing Yu, and Stanislav S. Zakharenko

SUPPLEMENTAL EXPERIMENTAL PROCEDURES

Rotarod Test

Motor coordination and balance were assessed using a rotarod (ROTOR-ROD™ System; San Diego Instruments). Mice were placed on a rotating rod (diameter 3.18 cm) in lanes that were 11.4-cm wide to maintain the animal in the same direction while the bar was rotating. The bar was 46 cm from the floor of the apparatus, and the bar's speed of rotation was gradually and linearly increased from 0 to 40 rpm over the 5-min trial. Mice were tested by using five trials per day for 2 consecutive days. Both the latency (s) and the distance (cm) at which the mice were able to maintain their balance on the bar were recorded automatically using the beam break technology.

Hot-plate Test

Nociception was tested using a hot-plate set (Columbus Instruments). Mice were placed on a 55°C hot plate, and the latency period to the first lift of the hind-limb response, including paw shake or licking, was recorded.

In Vivo Viral Injections

For *Drd2* overexpression or knockdown, previously described lentiviruses and AAVs (Chun et al., 2014) were used. Briefly, *AAV-CamKIIα-Drd2-EGFP* and *AAV-CamKIIα-EGFP* were used for the *Drd2* overexpression experiments, and lentiviral vectors *LV-U6-Drd2-EGFP* and *LV-U6-EGFP* were used for the *Drd2*-knockdown experiments. For DREADDs experiments, commercially available *AAV-hSyn-DIO-hM4Di-mCherry* (*hM4Di*) (UNC Vector Core; serotype 5; 6.7×10^{12} infection-forming units/mL) and *CAV2-Cre* (Institute of Molecular Genetics of Montpellier, France, and a gift from Dr. Darvas; 6×10^{12} particles/mL) were used. For in vivo viral injections, young adult mice were anesthetized with isoflurane in pure oxygen (2%–3% for induction and 1.0%–1.5% for maintenance). Mouse heads were then fixed in a stereotaxic device. Viruses were injected with a metal cannula (33 gauge; Plastics One). Briefly, an incision was made in the scalp, and a small hole was drilled for craniotomy. The following stereotaxic coordinates were used for in vivo injection: MGm (anterior-posterior, –3.16 mm; lateral ±2.0 mm, ventral, –3.0 mm) and LA (anterior-posterior, –1.4 mm; lateral, ±3.4 mm, ventral, –5.0 mm). After injections, incisions were sutured

and mice were allowed to recover before being returned to their holding cages. Experiments were performed approximately 3 weeks after AAV and CAV2 injections and 7 to 10 days after lentiviral injections.

Drug Administration

CNO (Enzo Life Sciences) was dissolved at 0.1 mg/mL in dH_2O and administered via an intraperitoneal injection (1.0 mg/kg) 0.5 h before the start of each behavioral test. Tamoxifen (100 μL, 40 mg/mL; Sigma) was injected intraperitoneally over 2 consecutive days.

Histologic Analysis

At the end of experiments, mice were deeply anesthetized and intracardially perfused with 4% paraformaldehyde in 0.1 mol/L phosphate buffer (pH 7.4), and brains were fixed overnight. Each brain was sliced (50 μm) coronally with a vibratome (Leica) through the rostrocaudal MGm/posterior intralaminar nucleus and the LA and stored in PBS. Virus injection needle tracks were identified with GFP, tdTomato, or mCherry expression, and DAPI (Invitrogen) or bright-field imaging was used as counterstain. Data from mice lacking histologic evidence were excluded from the analysis.

Western Blotting

Mouse brain tissues were lysed in ice-cold RIPA buffer [50 mM Tris-HCl (pH 7.4), 1% NP-40, 0.25% sodium deoxycholate, 150 mM NaCl, and 1 mM EDTA] that included protease and phosphatase inhibitor cocktail tablets (Roche). A total of 25 μg protein was loaded per lane. Sodium dodecyl sulfate/polyacrylamide gel electrophoresis, protein transfer to polyvinylidene difluoride membranes, and Western blotting were performed using standard techniques. The following primary antibodies were used: rabbit anti-DRD2 (Abcam, ab85367, 1:500), anti-DGCR8 (Proteintech, 10996-1-AP, 1:500), and mouse anti–β-actin (Sigma-Aldrich, A5316, 1:10,000). The following secondary antibodies were used: anti-rabbit (LI-COR Biosciences, 926-68021; 1:15,000) and anti-mouse (LI-COR Biosciences, 926-32212, 1:15,000) antibodies conjugated to IR dye 680 or 800, respectively. Blots were imaged and quantified using the Odyssey CLx infrared imaging system (LI-COR Biosciences).

Table S1. Comparison of behavioral and electrophysiological phenotypes in 22q11DS mouse mutants and following disruption of synaptic transmission at thalamoamygdala projections. Related to Figures 1-4, 7.

**Genotype or treatment**[a]	**Fear conditioning (%)**	**Active avoidance**[b] **(%)**	**Basal synaptic transmission**[b] **(%)**	**Paired-pulse depression**[b] **(%)**
Df(16)1/+ mice	–29.4 ± 8.8 *	–26.0 ± 5.8 *	–38.6 ± 7.1 *	–29.4 ± 4.8 *
cDgcr8$^{+/-}$ mice	–23.6 ± 7.9 *	6.3 ± 9.5	–23.5 ± 6.0 *	–10.4 ± 2.7 *
cDgcr8$^{-/-}$ mice	–40.4 ± 7.0 *	–42.3 ± 7.8 *	–65.8 ± 5.2 *	–39.2 ± 2.3 *
Drd2 OE in WT mice	–45.1 ± 7.4 *	–45.7 ± 9.1 *	–77.3 ± 12.8 *	–59.2 ± 5.5 *
Retro-DREADD in WT mice	–26.2 ± 7.0 *	–22.4 ± 8.6 *	–51.9 ± 8.2 *	–33.5 ± 4.5 *

[a] Relative changes for each genotype or treatment group were compared to their respective controls. The *cDgcr8*$^{+/-}$ and *cDgcr8*$^{-/-}$ mice; *Gbx2*^{CreER};*Dgcr8*$^{fl/+}$ and *Gbx2*^{CreER};*Dgcr8*$^{fl/fl}$ mice, respectively. *Drd2* OE: mice with overexpression of *Drd2* in the auditory thalamus. Retro-DREADD: mice injected with *CAV2-Cre* into the lateral amygdala and *hM4Di* into the auditory thalamus and treated with CNO. *$p < 0.05$.

[b]For active avoidance and electrophysiological experiments, values were calculated as normalized to respective WT averages among sessions, stimulating intensities, and interpulse intervals. Data are represented as mean ± SEM.

SUPPLEMENTARY FIGURES

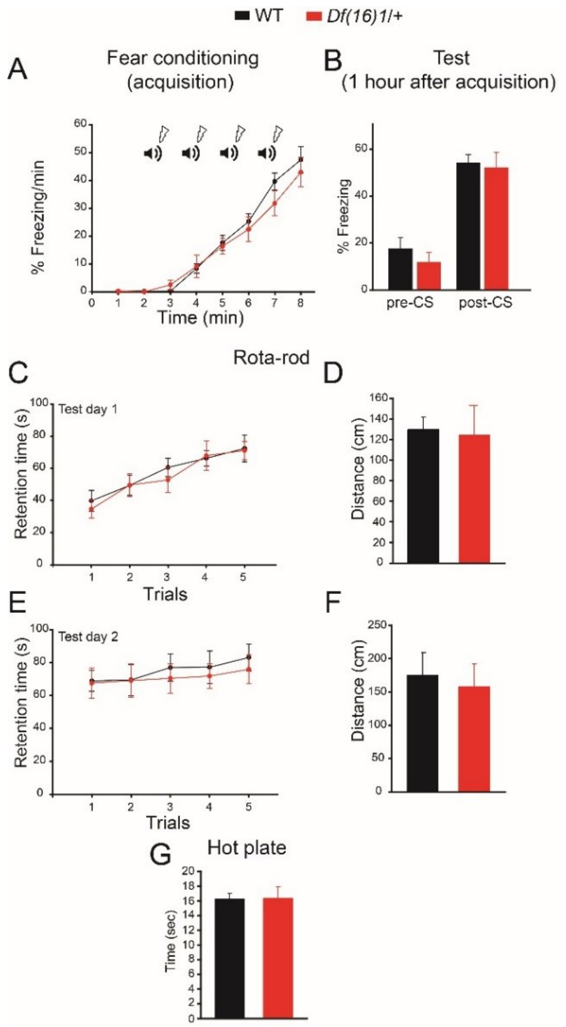

Figure S1. Normal Learning of the Fear Conditioning Task, Locomotion, and Pain Threshold in 22q11DS Mice. Related to Figure 1.

(A) Freezing behavior on training day 1 in WT (n=14) and *Df(16)1*/+ (n=14) animals. Two-way repeated-measures ANOVA; $F_{1,7}$=0.47, p=0.499. Symbols for the conditioned stimuli (CS; tones) and unconditioned stimuli (US; foot shocks) are shown.

(B) Freezing behavior measured in the distinct context 1 hr after fear conditioning in WT and *Df(16)1/*+ mice before tone presentation (pre–CS). WT: 14 mice, *Df(16)1/*+: 14 mice. Mann-Whitney rank sum test: *U*=72.5, p=0.25. Freezing behavior during tone presentation (post-CS): two-tailed Student's *t*-test: t_{26}=0.275, p=0.785.

(C–F) Retention time (C, E) and total distance (D, F) in the rotarod test performed on 2 consecutive days in WT (n=12) and *Df(16)1/*+ (n=11) animals. (C, E) Two-way repeated measures ANOVA. C: $F_{1,4}$=0.131, p=0.721; E: $F_{1,4}$=0.177, p=0.678. (D, F) Mann-Whitney rank sum test. D: *U*=50, p=0.34; F: *U*=56, p=0.559.

(G) Response latency (first lift of the hind limb) in the hot plate test in WT (n=12) and *Df(16)1/*+ (n=11) animals. Mann-Whitney rank sum test: *U*=57.5, p=0.619. Data are represented as mean ± SEM.

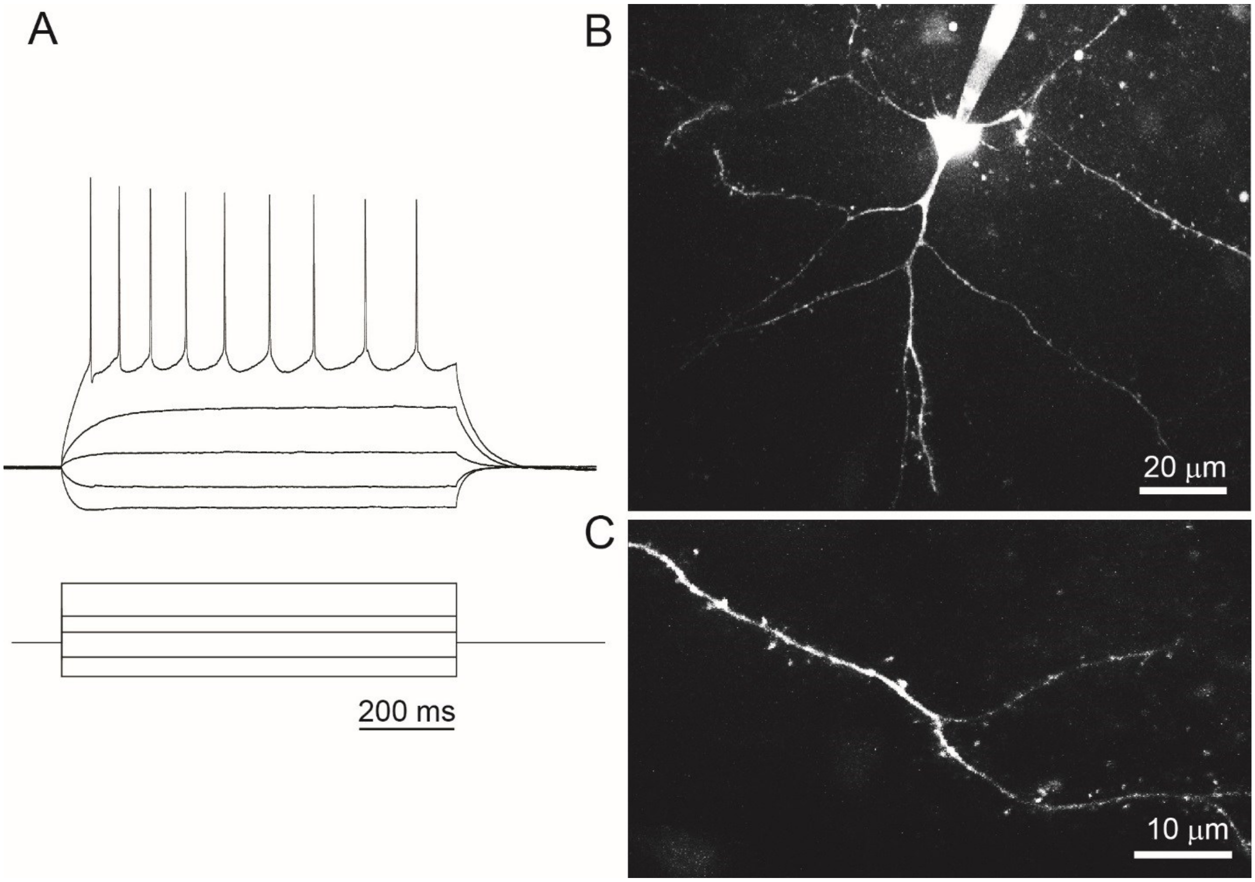

Figure S2. Identification of the Excitatory Neurons in the Lateral Amygdala. Related to Figure 1.
(A) Current-clamp recordings show action potential accommodation in an excitatory LA neuron during the injection of a depolarizing current. (Bottom) A protocol depicting injections of –80 pA, –40 pA, 20 pA, 60 pA, and 140 pA currents into an LA neuron.
(B, C) Fluorescence images of an LA excitatory neuron (B) and its dendrite (C).

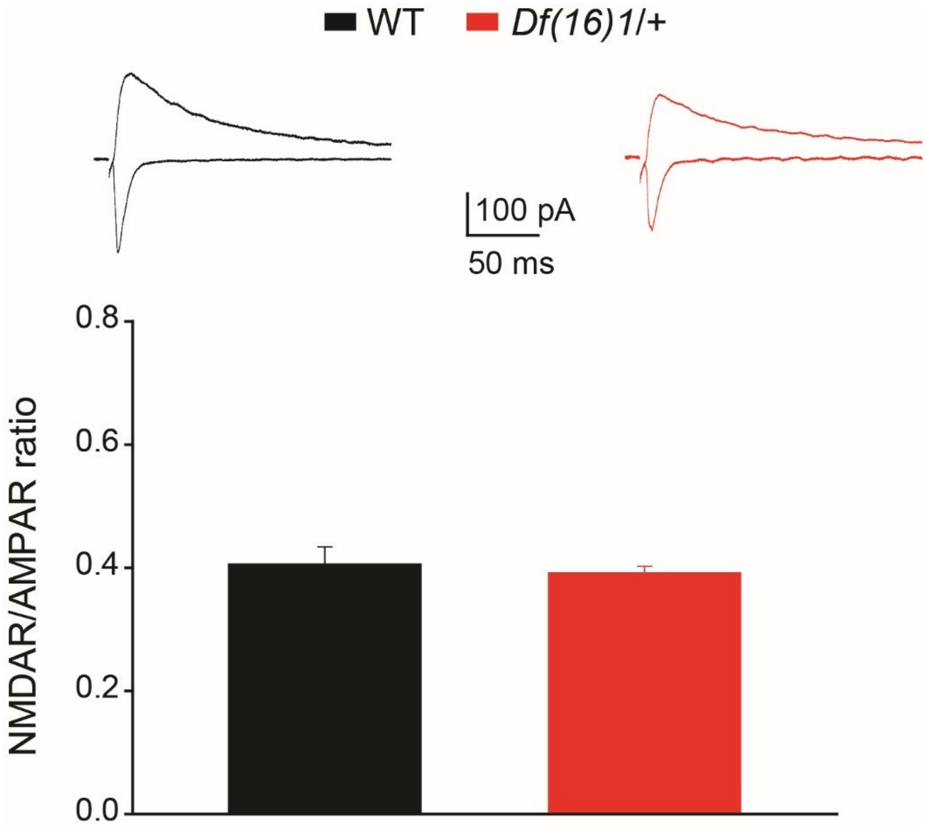

Figure S3. Normal Postsynaptic Function at Thalamo-LA Projections of 22q11DS Mice. Related to Figure 1. Mean NMDAR/AMPAR ratios at thalamo-LA projections of WT (5 neurons, 3 mice) and *Df(16)1/+* (8 neurons, 4 mice) littermates. Two-tailed *t* test: t_{11}=0.556, p=0.589. Picrotoxin (100 µM) was present in the ACSF. Insets show representative NMDAR-mediated EPSCs (recorded at +40 mV) and AMPAR-mediated EPSCs (recorded at −70 mV). Data are represented as mean ± SEM.

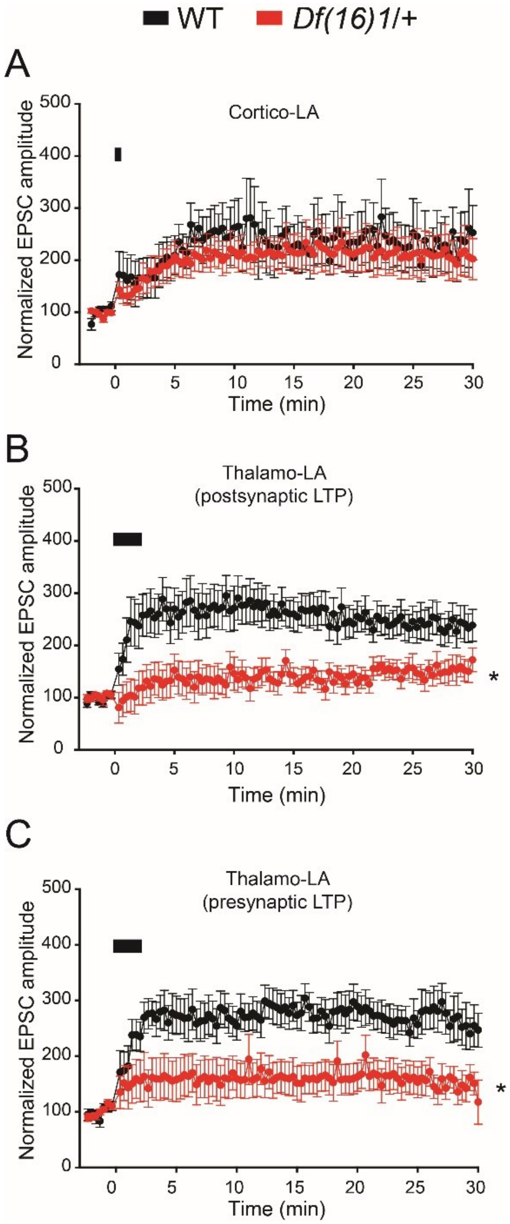

Figure S4. Impaired Thalamo-LA LTP but not Cortico-LA LTP in 22q11DS Mice. Related to Figure 1.

(A-C) Mean excitatory postsynaptic currents (EPSCs) were normalized to initial baseline at cortico-LA (A) and thalamo-LA (B, C) projections, before and after induction of LTP in WT (A: 9 neurons, 3 mice; B: 15 neurons, 8 mice; C: 10 neurons, 5 mice) or *Df(16)1/+* (A: 22 neurons, 8 mice; B: 12 neurons, 5 mice; C: 9 neurons, 5 mice) animals. A: Mann-Whitney rank sum test: U=65, p=0.145, B: two-tailed t test: t_{25}=2.358, *p=0.026, C: two-tailed t test: t_{17}=2.87, *p=0.01. Postsynaptically (B) or presynaptically (C) expressed LTP was induced in thalamo-LA projections by using previously established induction protocols (see Methods) (Shin et al., 2010). Data are represented as mean ± SEM. Solid bars, stimulus-induction protocols.

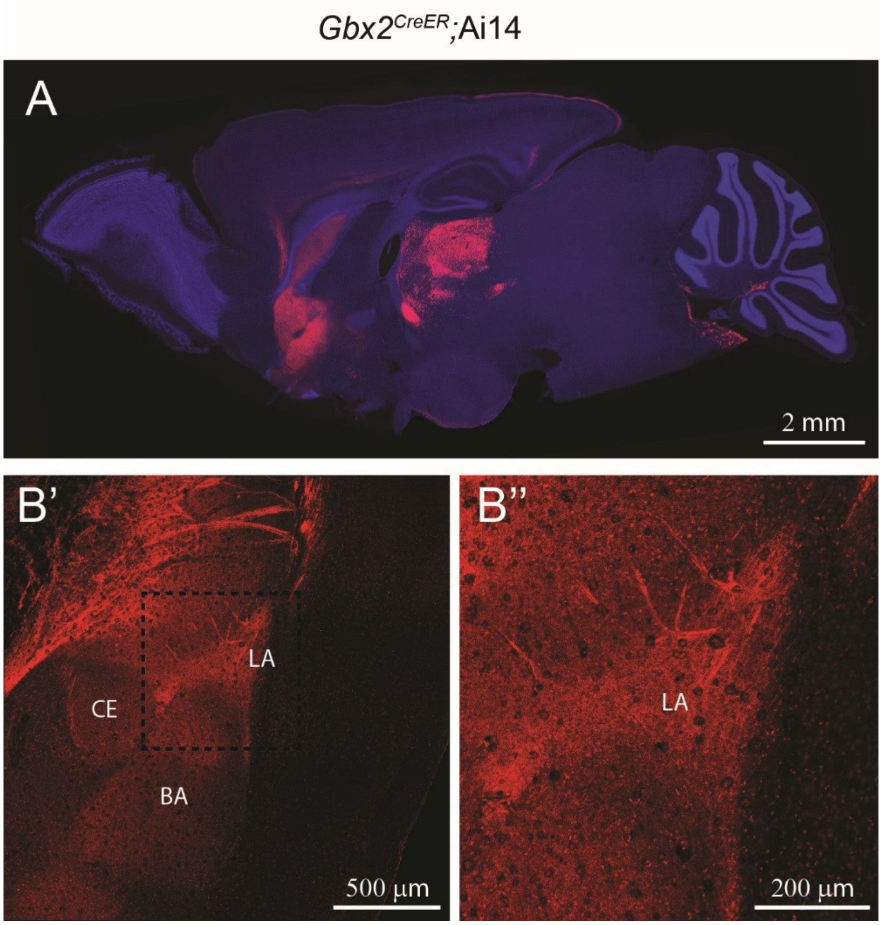

Figure S5. Cre Recombinase Expression in Thalamic Neurons and Their Axonal Projections in *Gbx2^{CreER}*;Ai14 Mice. Related to Figure 2.

(A) Fluorescence image of tdTomato in a sagittal brain section (lateral 0.48 mm) of a mature *Gbx2^{CreER}*;Ai14 mouse 14 days after an intraperitoneal tamoxifen injection. The section is counterstained with DAPI to label nuclei.

(B') The tdTomato-labeled thalamic axonal projections in the LA.

(B'') A higher magnification of the inset in B'. BA, basolateral amygdaloid nucleus; CE, central amygdaloid nucleus; LA, lateral amygdala.

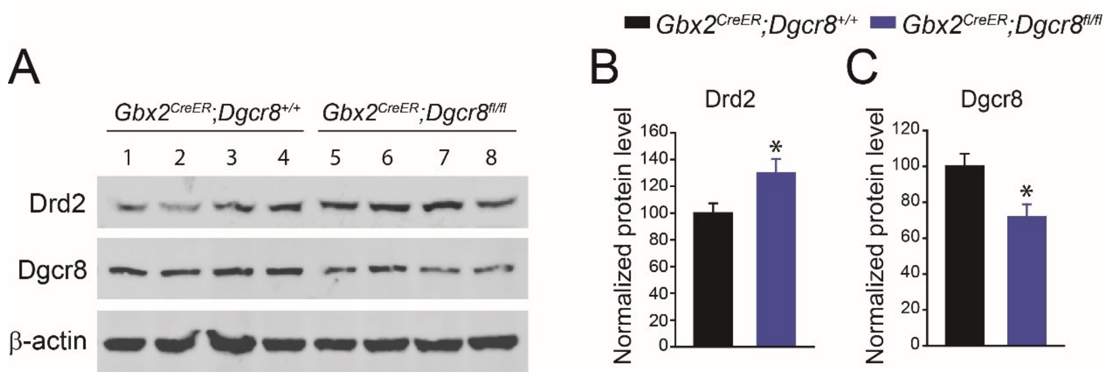

Figure S6. Drd2 and Dgcr8 Protein Levels in the Auditory Thalamus of *cDgcr8* KO Mice. Related to Figure 2.

(A) Representative Western blot images of Drd2 (50 kDa) and Dgcr8 (86 kDa) proteins in the auditory thalamus of WT (*Gbx2*^{CreER}*;Dgcr8*$^{+/+}$) and *cDgcr8* KO (*Gbx2*^{CreER}*;Dgcr8*$^{fl/fl}$) mice. WT: 4 mice, lanes 1-4; *cDgcr8* KO: 4 mice, lanes 5-8.

(B, C) Relative Drd2 and Dgcr8 protein levels normalized to respective β-actin levels in the auditory thalamus of WT and *cDgcr8* KO animals. Two-tailed *t* test; Drd2 (run in duplicates): t_{14}=2.344, *p=0.034; Dgcr8: t_6=2.805, *p=0.03. Data are represented as mean ± SEM.

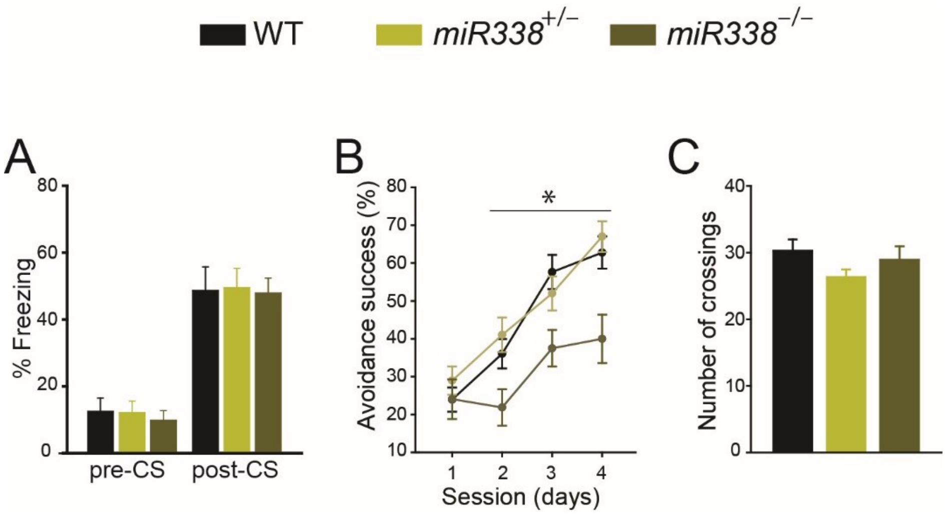

Figure S7. Effect of *miR338* Deletion on Associative Fear Learning. Related to Figure 3.

(A) Freezing behavior in WT (8 mice), *miR338*$^{+/–}$ (13 mice), and *miR-338*$^{–/–}$ (10 mice) littermates during the fear conditioning test. Kruskal-Wallis one-way ANOVA, pre-CS: H_2=0.362, p=0.834; post-CS: F_2=0.003, p=0.978.

(B) Performance of WT (19 mice), *miR338*$^{+/–}$ (20 mice) and *miR-338*$^{–/–}$ (16 mice) littermates in the active avoidance test. Two-way repeated measures ANOVA: $F_{2,3}$=9.940, *p<0.001. Post-hoc: WT vs. *miR338*$^{+/–}$, p=0.593; WT vs. *miR338*$^{–/–}$, *p =0.001.

(C) Total number of crossings between compartments. One-way ANOVA: F_2=1.637, p=0.204. Data are represented as mean ± SEM.

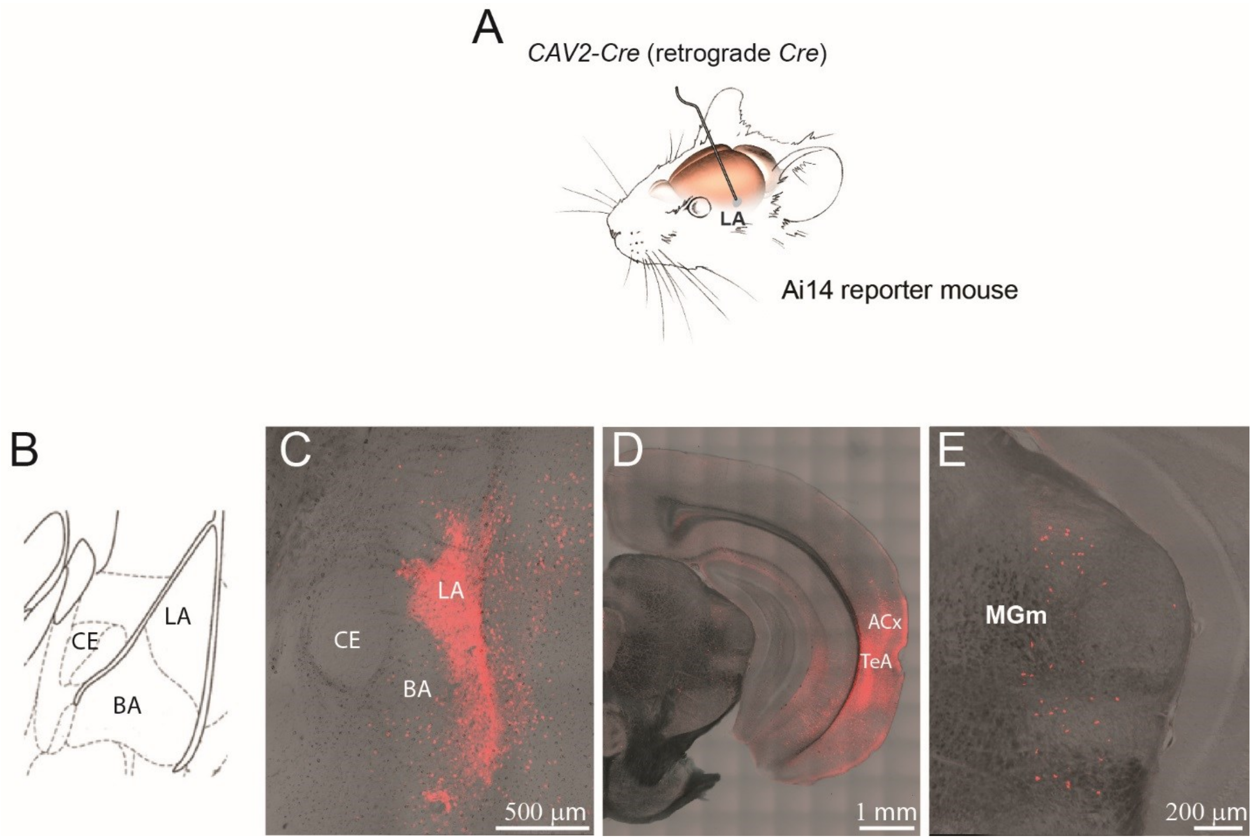

Figure S8. Verification of the Retro-DREADDs Approach in Ai14 Mice. Related to Figure 7.

(A) *CAV2-Cre* viruses were injected into the LA of an Ai14 reporter mouse.

(B) A histologic plate of the amygdaloid nuclei complex in a coronal section at Bregma −1.82 mm, adapted from (Paxinos and Franklin, 2001).

(C–E) Representative images of coronal sections 3 weeks after the *CAV2-Cre* injection into the LA. The expression of tdTomato in the LA depicts the specific targeting of *CAV2–Cre* into the LA (C) and its retrograde infection of neurons in the auditory cortex (ACx), temporal association cortex (TeA) (D), and the auditory thalamus (including the MGm nucleus) (E). BA, basolateral amygdaloid nucleus; CE, central amygdaloid nucleus; LA, lateral amygdala.

Reference List

Chun, S, Westmoreland, JJ, Bayazitov, IT, Eddins, D, Pani, AK, Smeyne, RJ, Yu, J, Blundon, JA, and Zakharenko, SS (2014). Specific disruption of thalamic inputs to the auditory cortex in schizophrenia models. Science. *344*, 1178-1182.

Paxinos,G, and Franklin,KBJ (2001). Mouse Brain in Stereotaxic Coordinates (New York: Academic Press).

Shin, RM, Tully, K, Li, Y, Cho, JH, Higuchi, M, Suhara, T, and Bolshakov, VY (2010). Hierarchical order of coexisting pre- and postsynaptic forms of long-term potentiation at synapses in amygdala. Proc. Natl. Acad. Sci. U. S. A *107*, 19073-19078.
